# Supplementary material for: Targeting Grb2 SH3 Domains with Affimer Proteins Provides Novel Insights into Ras Signalling Modulation
Source: Biomolecules. 2024 Aug 22;14(8):1040. doi: 10.3390/biom14081040 (PMC11352564; doi:10.3390/biom14081040)
Supplement: Supplementary file 1 [file biomolecules-14-01040-s001.zip › Figure S1/Figure 3 uncropped.pptx]

## Slide 1
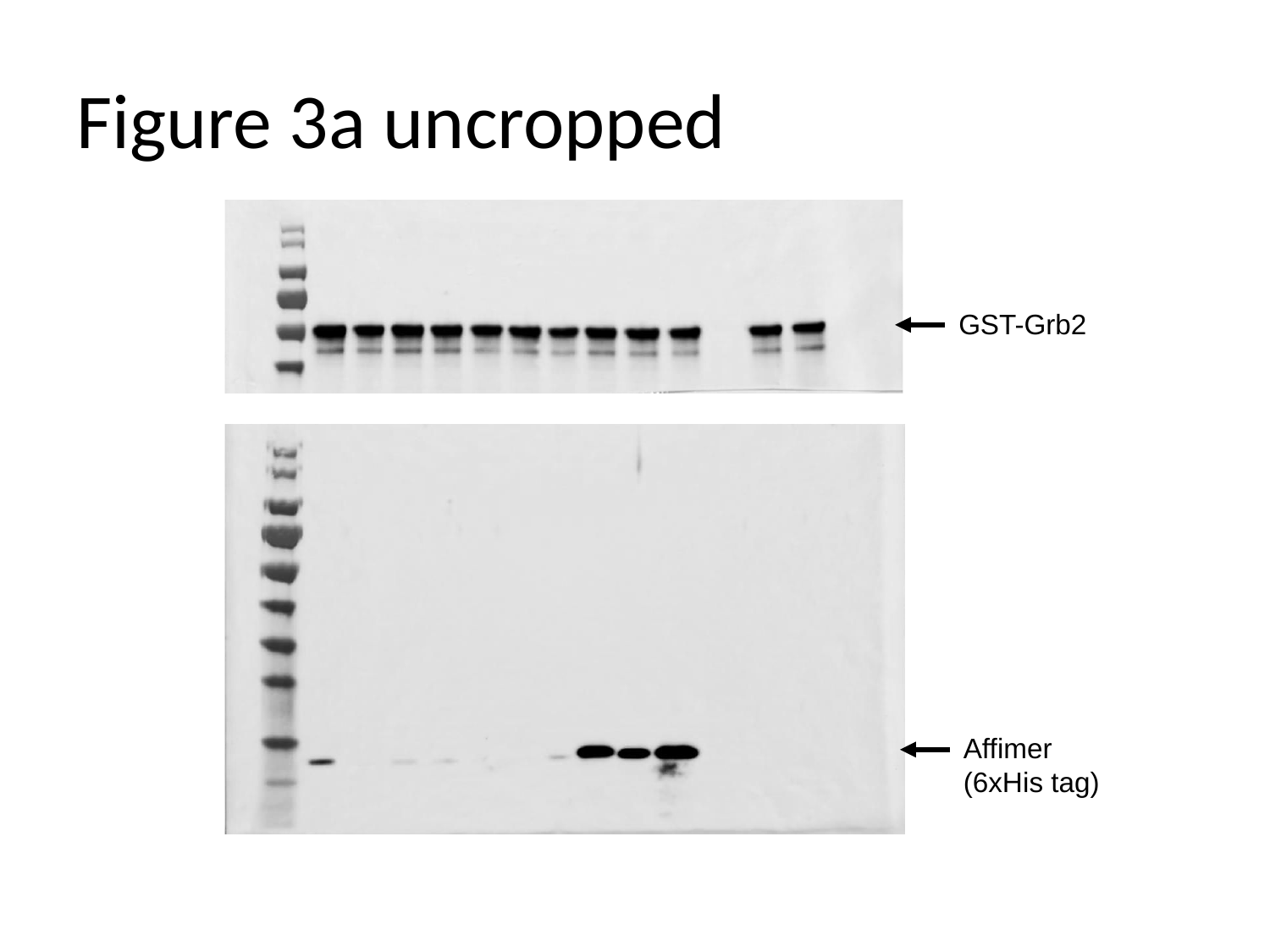

# Figure 3a uncropped
GST-Grb2
Affimer
(6xHis tag)

## Slide 2
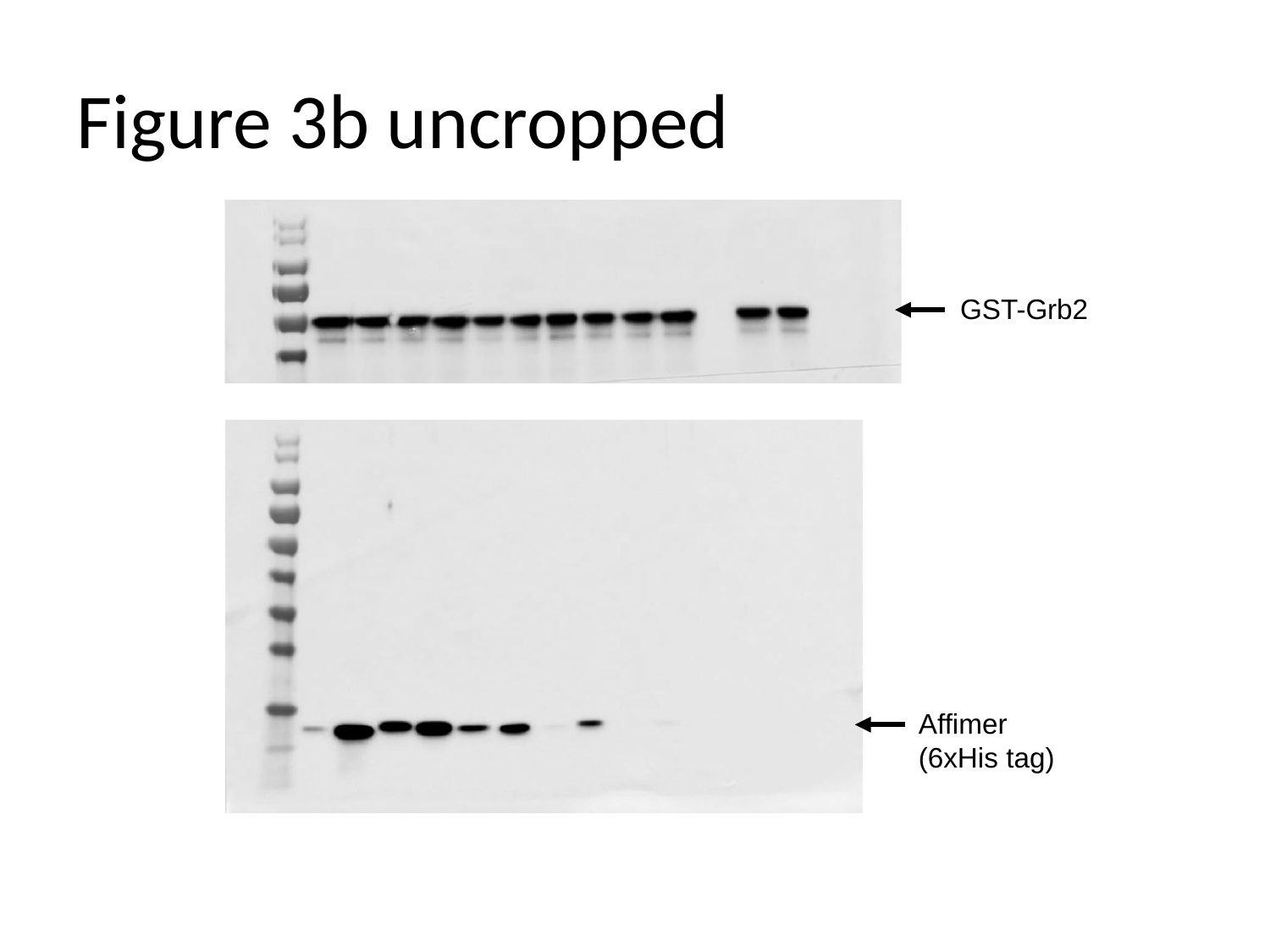

# Figure 3b uncropped
GST-Grb2
Affimer
(6xHis tag)

## Slide 3
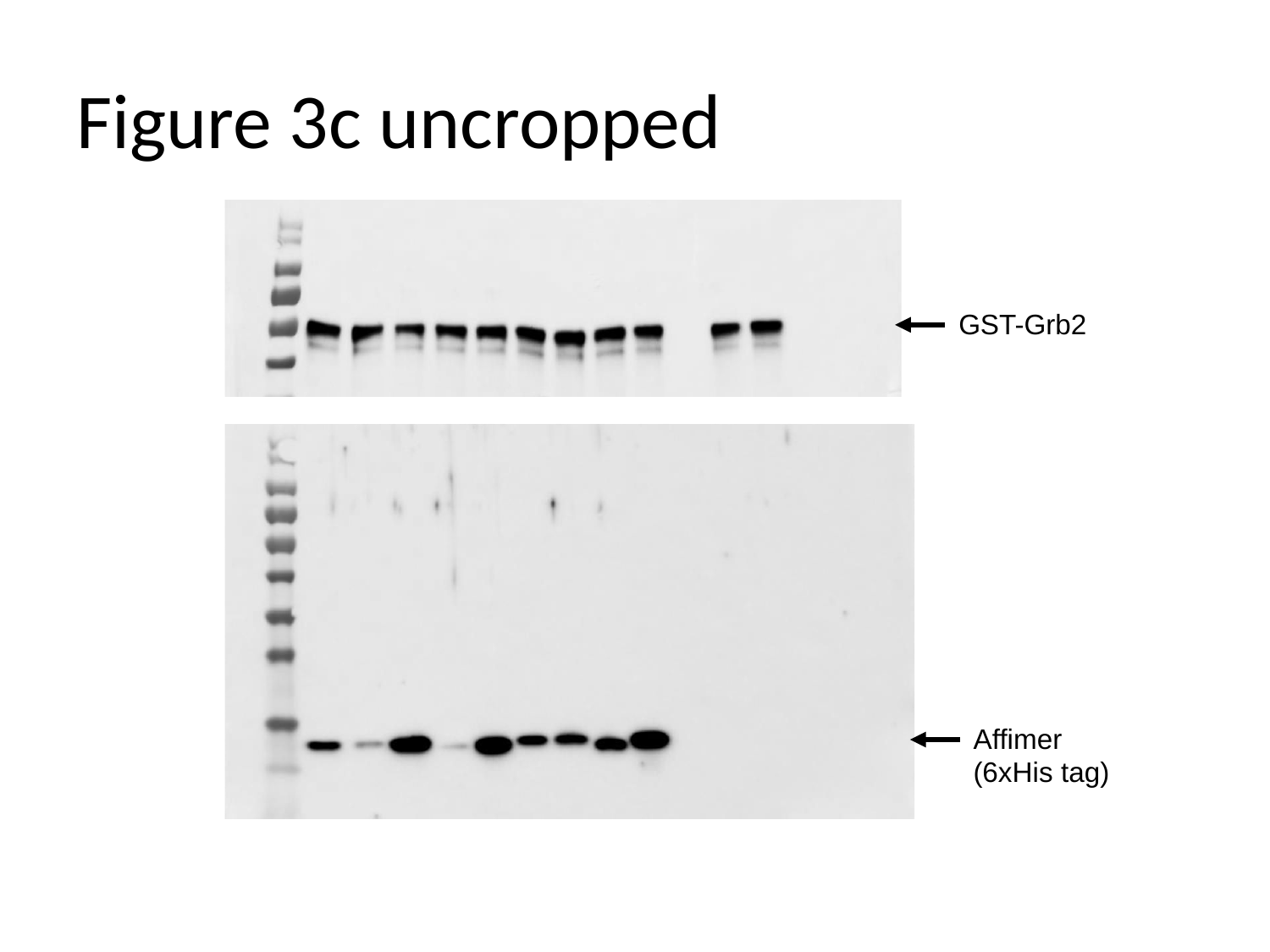

# Figure 3c uncropped
GST-Grb2
Affimer
(6xHis tag)
