# Supplementary material for: Targeting Grb2 SH3 Domains with Affimer Proteins Provides Novel Insights into Ras Signalling Modulation
Source: Biomolecules. 2024 Aug 22;14(8):1040. doi: 10.3390/biom14081040 (PMC11352564; doi:10.3390/biom14081040)
Supplement: Supplementary file 1 [file biomolecules-14-01040-s001.zip › Figure S3/Figure S3 Pull-down from HEK-293.pptx]

## Slide 1
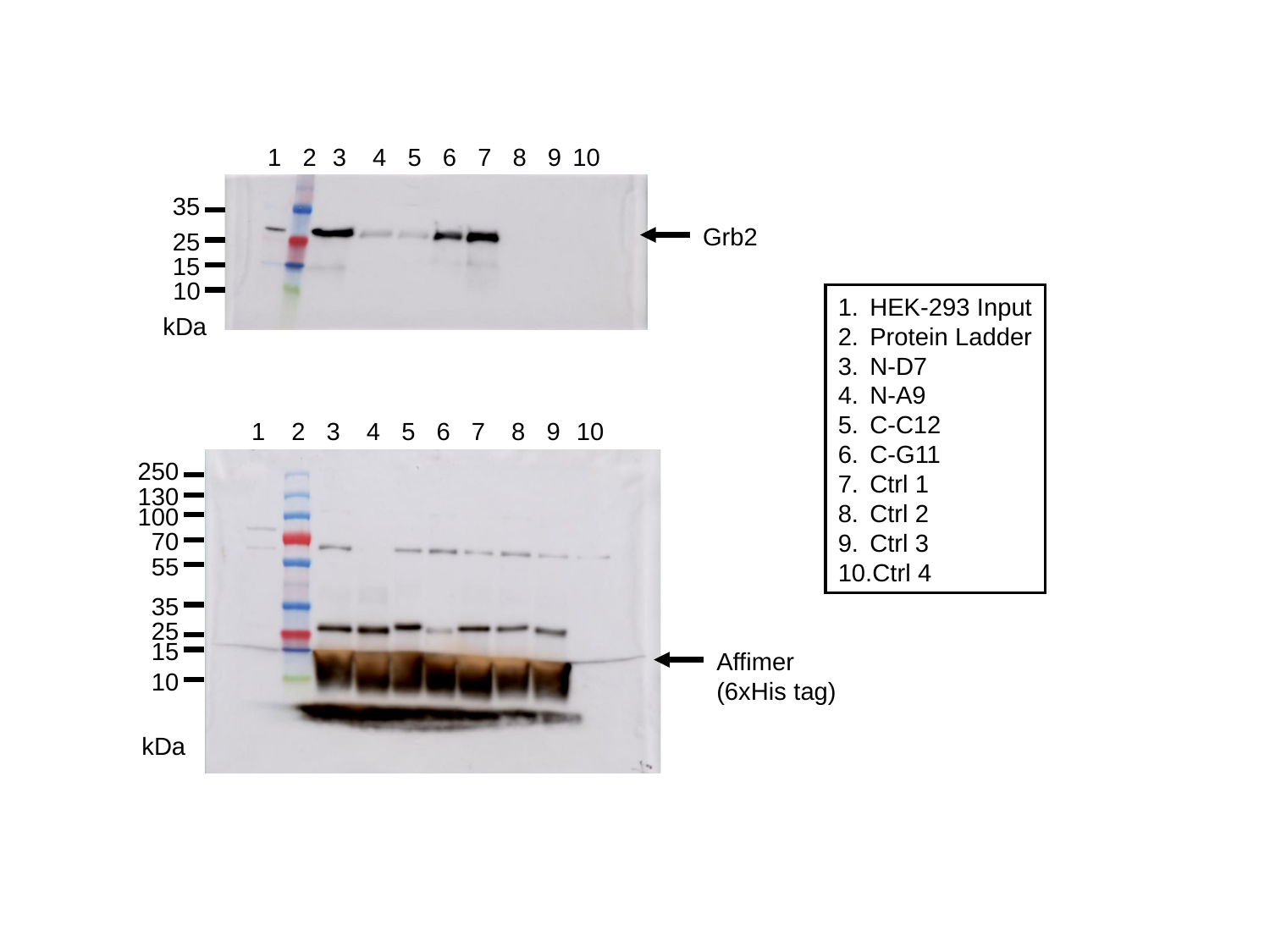

1
2
3
4
5
6
7
8
9
10
35
Grb2
25
15
10
kDa
HEK-293 Input
Protein Ladder
N-D7
N-A9
C-C12
C-G11
Ctrl 1
Ctrl 2
Ctrl 3
Ctrl 4
1
2
3
4
5
6
7
8
9
10
250
130
100
70
55
35
25
15
Affimer
(6xHis tag)
10
kDa

## Slide 2
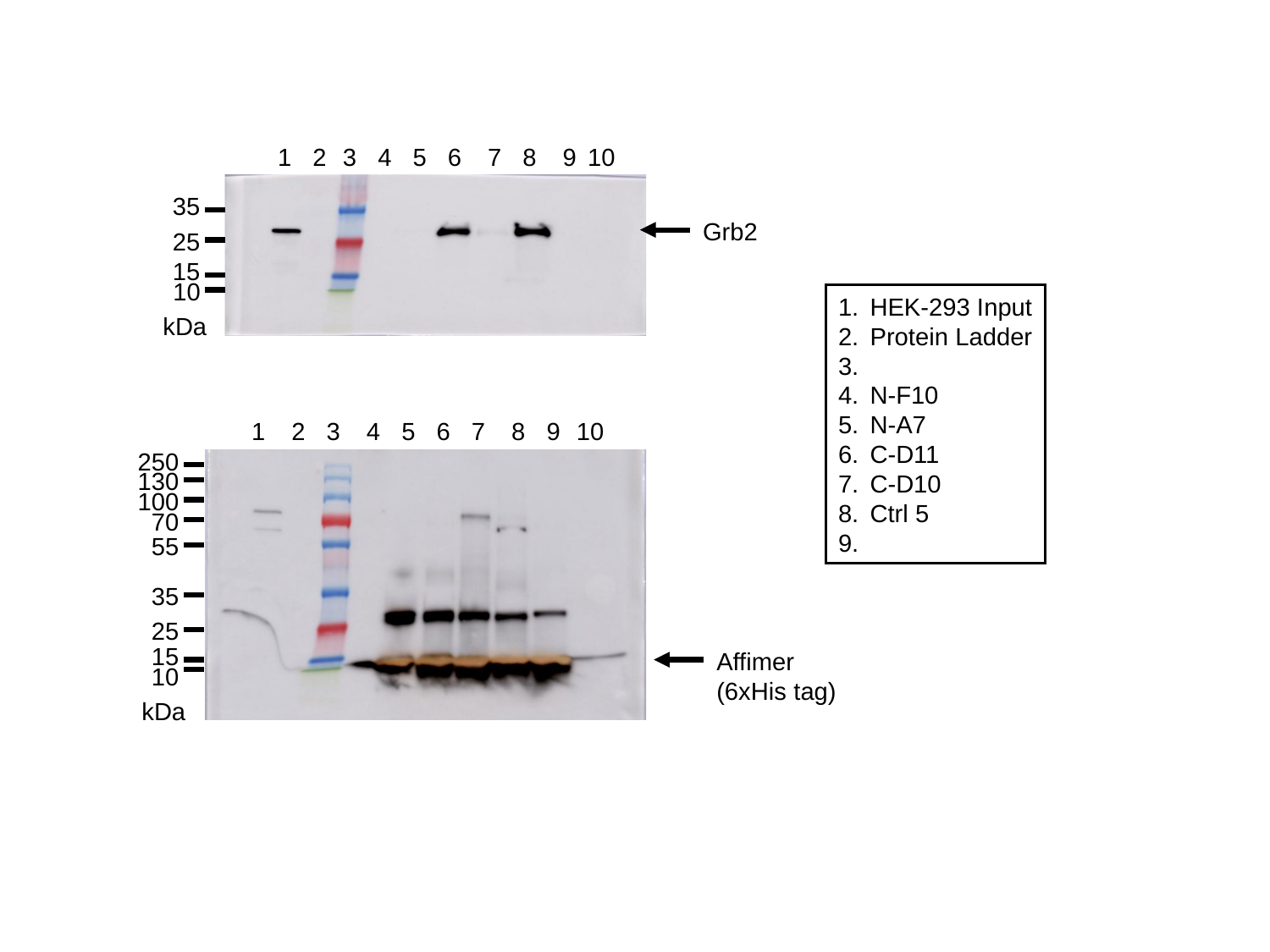

1
2
3
4
5
6
7
8
9
10
35
25
15
10
kDa
Grb2
HEK-293 Input
Protein Ladder
N-F10
N-A7
C-D11
C-D10
Ctrl 5
1
2
3
4
5
6
7
8
9
10
250
130
100
70
55
35
25
15
10
Affimer
(6xHis tag)
kDa

## Slide 3
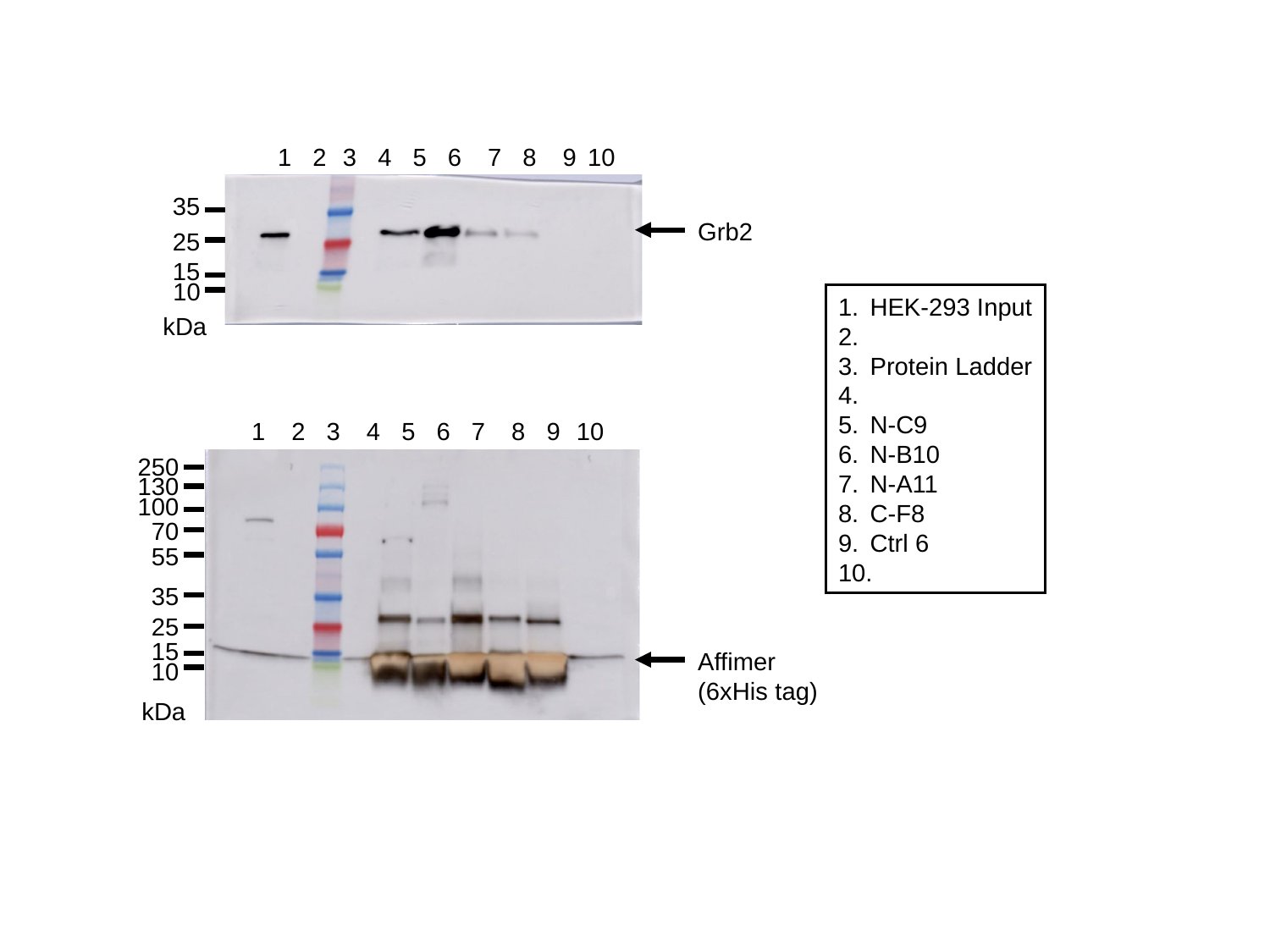

1
2
3
4
5
6
7
8
9
10
35
25
15
10
kDa
Grb2
HEK-293 Input
Protein Ladder
N-C9
N-B10
N-A11
C-F8
Ctrl 6
1
2
3
4
5
6
7
8
9
10
250
130
100
70
55
35
25
15
10
kDa
Affimer
(6xHis tag)
