# Supplementary material for: Targeting Grb2 SH3 Domains with Affimer Proteins Provides Novel Insights into Ras Signalling Modulation
Source: Biomolecules. 2024 Aug 22;14(8):1040. doi: 10.3390/biom14081040 (PMC11352564; doi:10.3390/biom14081040)
Supplement: Supplementary file 1 [file biomolecules-14-01040-s001.zip › Figure S4/Figure 8a & 8b uncropped.pptx]

## Slide 1
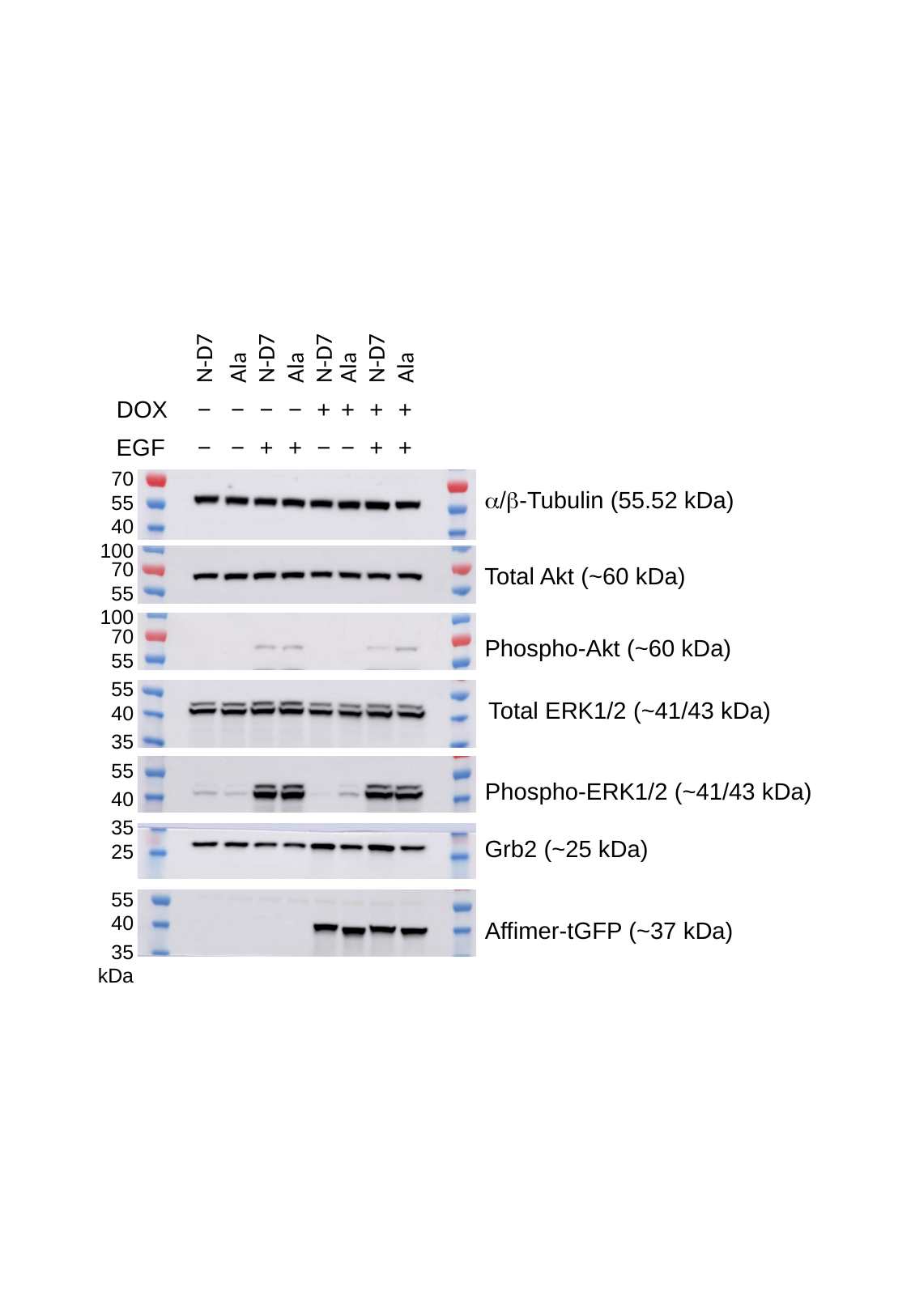

N-D7
N-D7
N-D7
N-D7
Ala
Ala
Ala
Ala
DOX
−
−
−
−
+
+
+
+
EGF
−
−
+
+
−
−
+
+
70
a/b-Tubulin (55.52 kDa)
55
40
100
70
Total Akt (~60 kDa)
55
100
70
Phospho-Akt (~60 kDa)
55
55
Total ERK1/2 (~41/43 kDa)
40
35
55
Phospho-ERK1/2 (~41/43 kDa)
40
35
Grb2 (~25 kDa)
25
55
40
Affimer-tGFP (~37 kDa)
35
kDa

## Slide 2
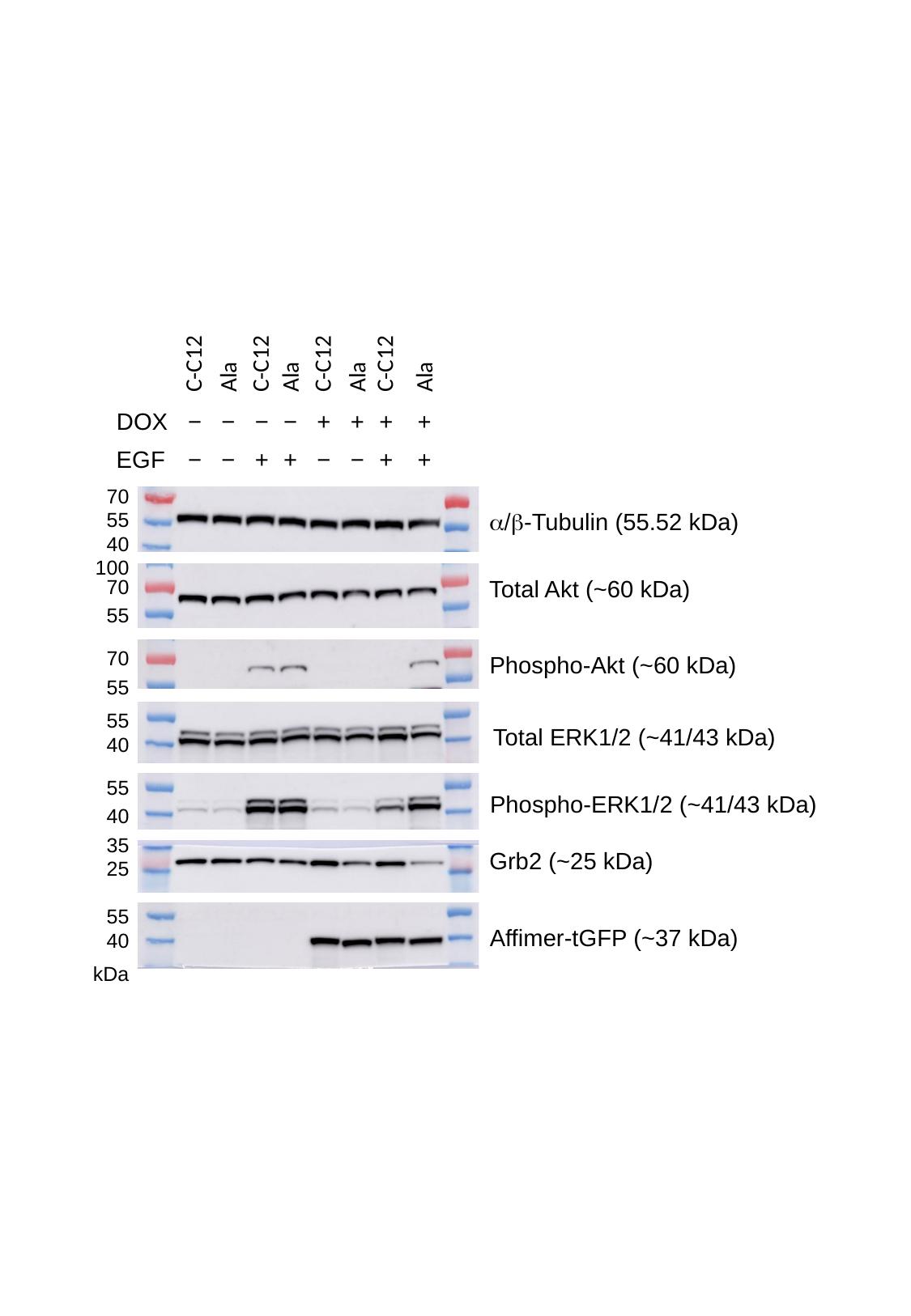

C-C12
C-C12
C-C12
C-C12
Ala
Ala
Ala
Ala
DOX
−
−
−
−
+
+
+
+
EGF
−
−
+
+
−
−
+
+
70
55
a/b-Tubulin (55.52 kDa)
40
100
70
Total Akt (~60 kDa)
55
70
Phospho-Akt (~60 kDa)
55
55
Total ERK1/2 (~41/43 kDa)
40
55
Phospho-ERK1/2 (~41/43 kDa)
40
35
Grb2 (~25 kDa)
25
55
Affimer-tGFP (~37 kDa)
40
kDa

## Slide 3
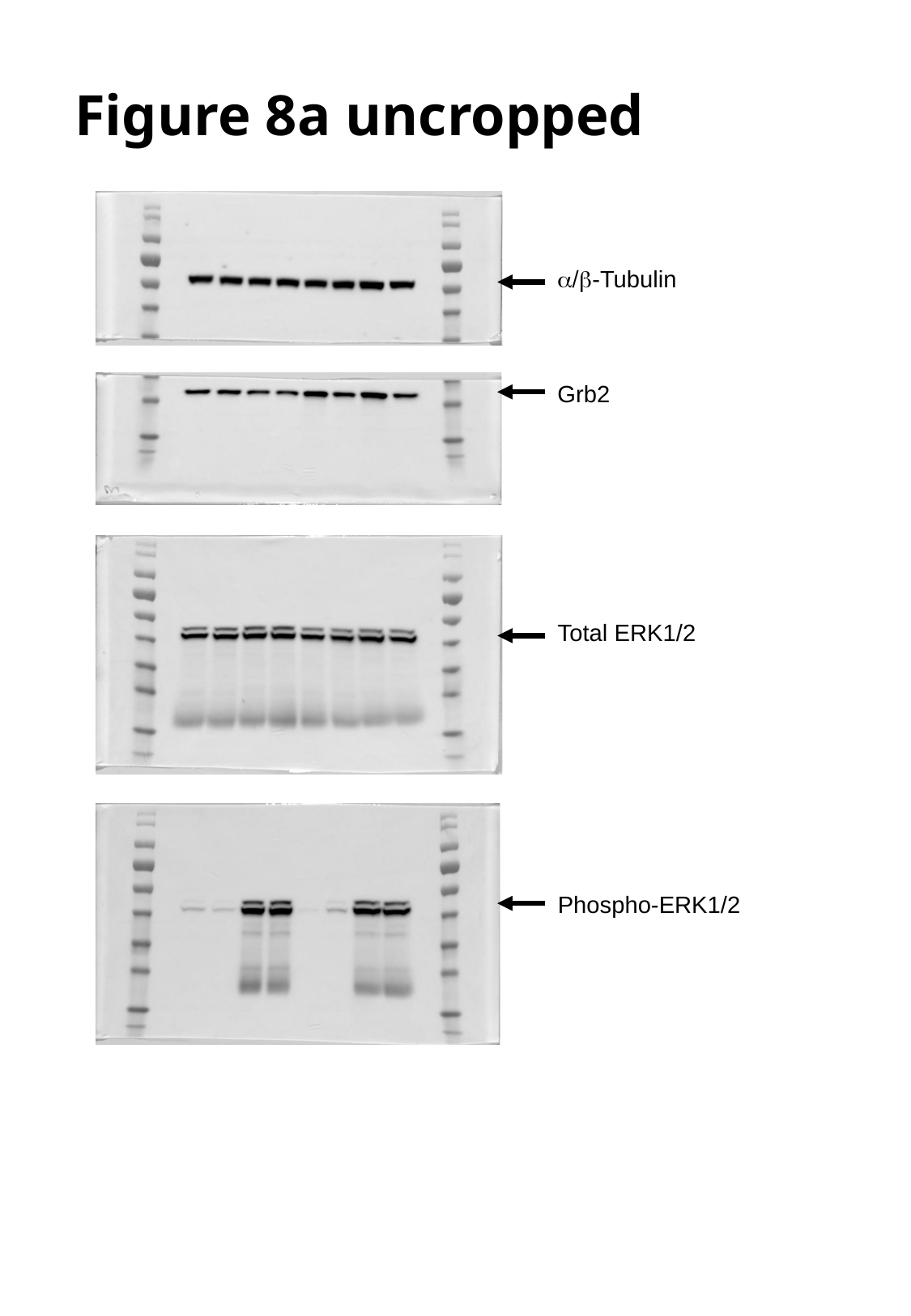

# Figure 8a uncropped
a/b-Tubulin
Grb2
Total ERK1/2
Phospho-ERK1/2

## Slide 4
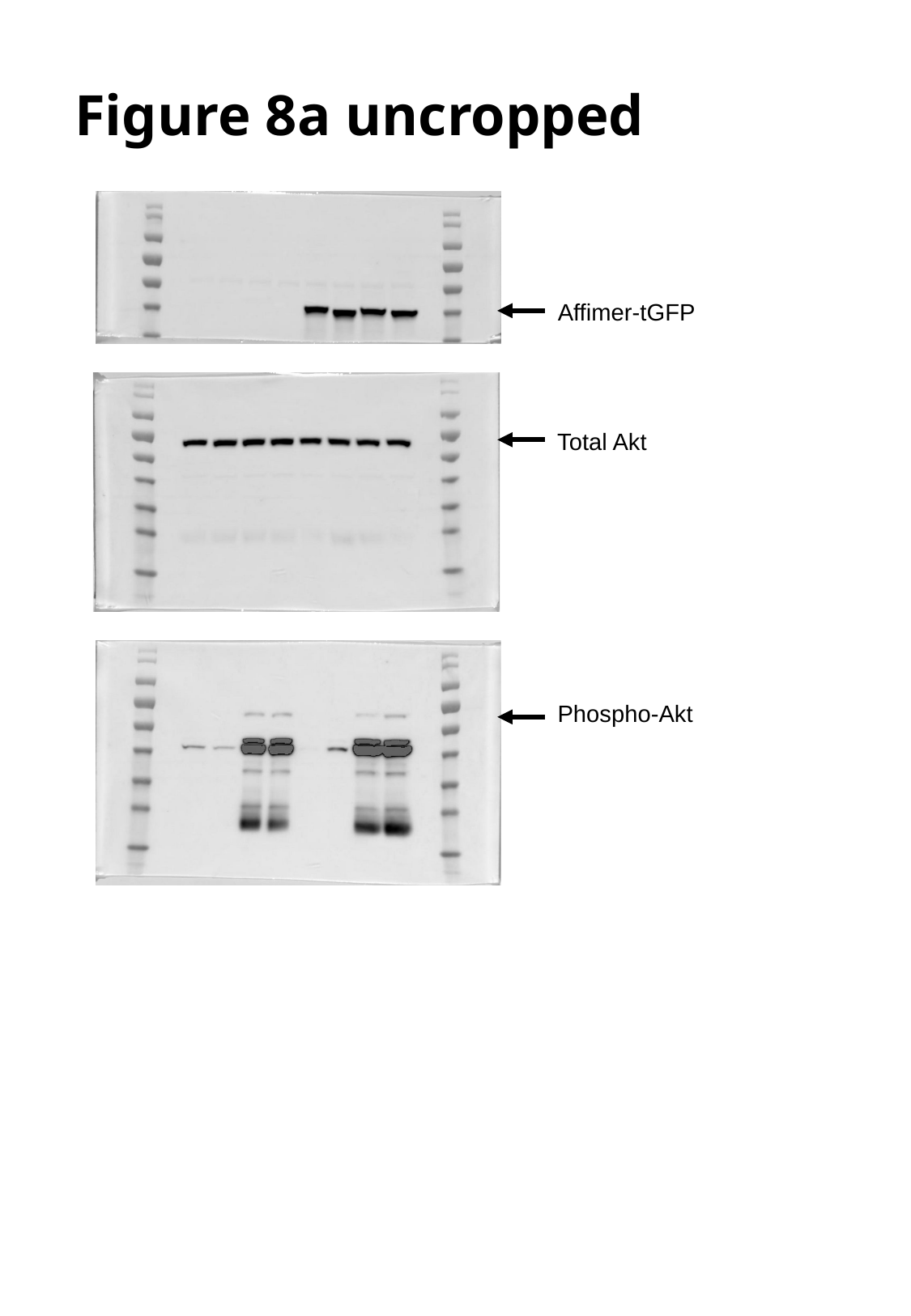

# Figure 8a uncropped
Affimer-tGFP
Total Akt
Phospho-Akt

## Slide 5
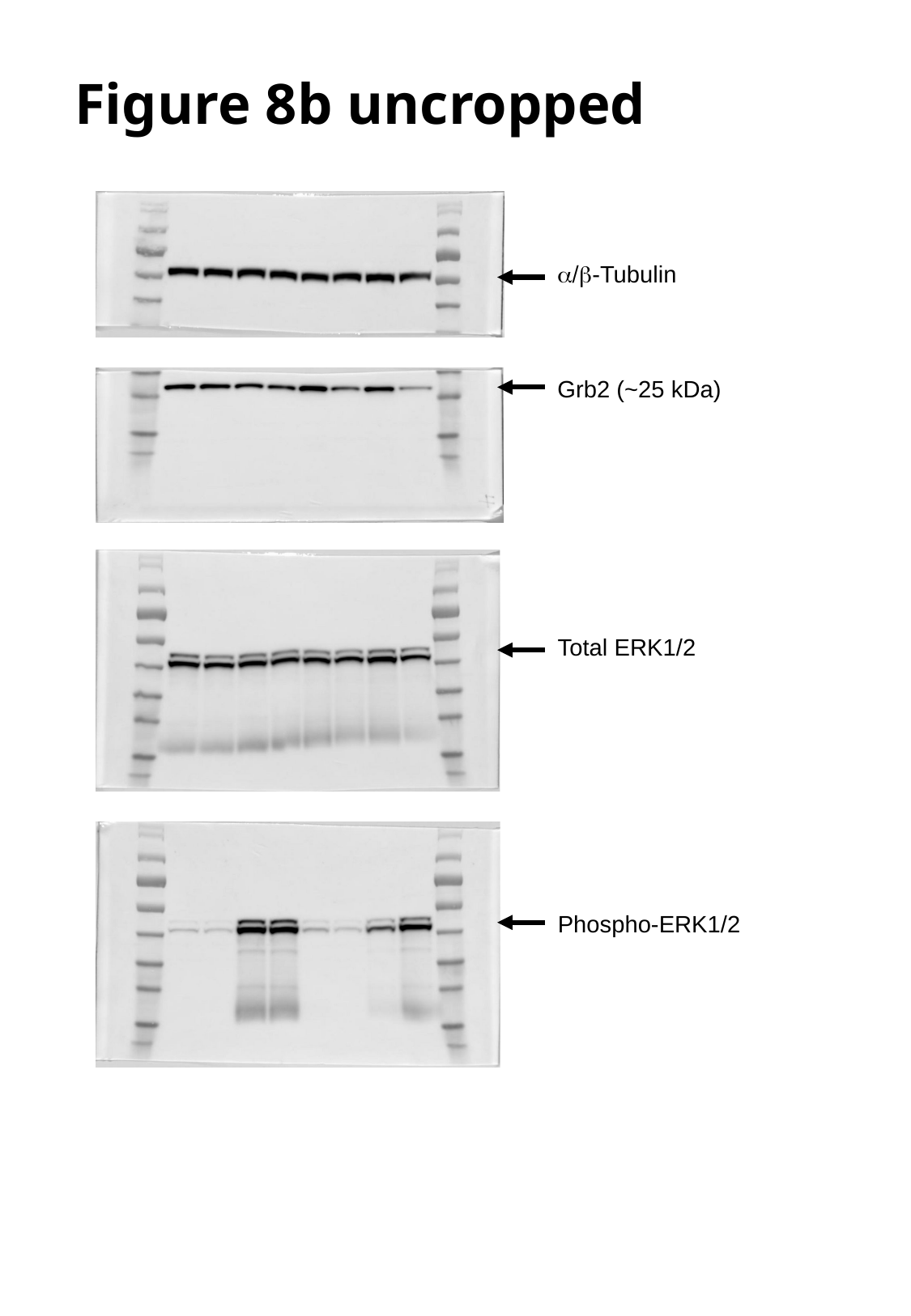

Figure 8b uncropped
a/b-Tubulin
Grb2 (~25 kDa)
Total ERK1/2
Phospho-ERK1/2

## Slide 6
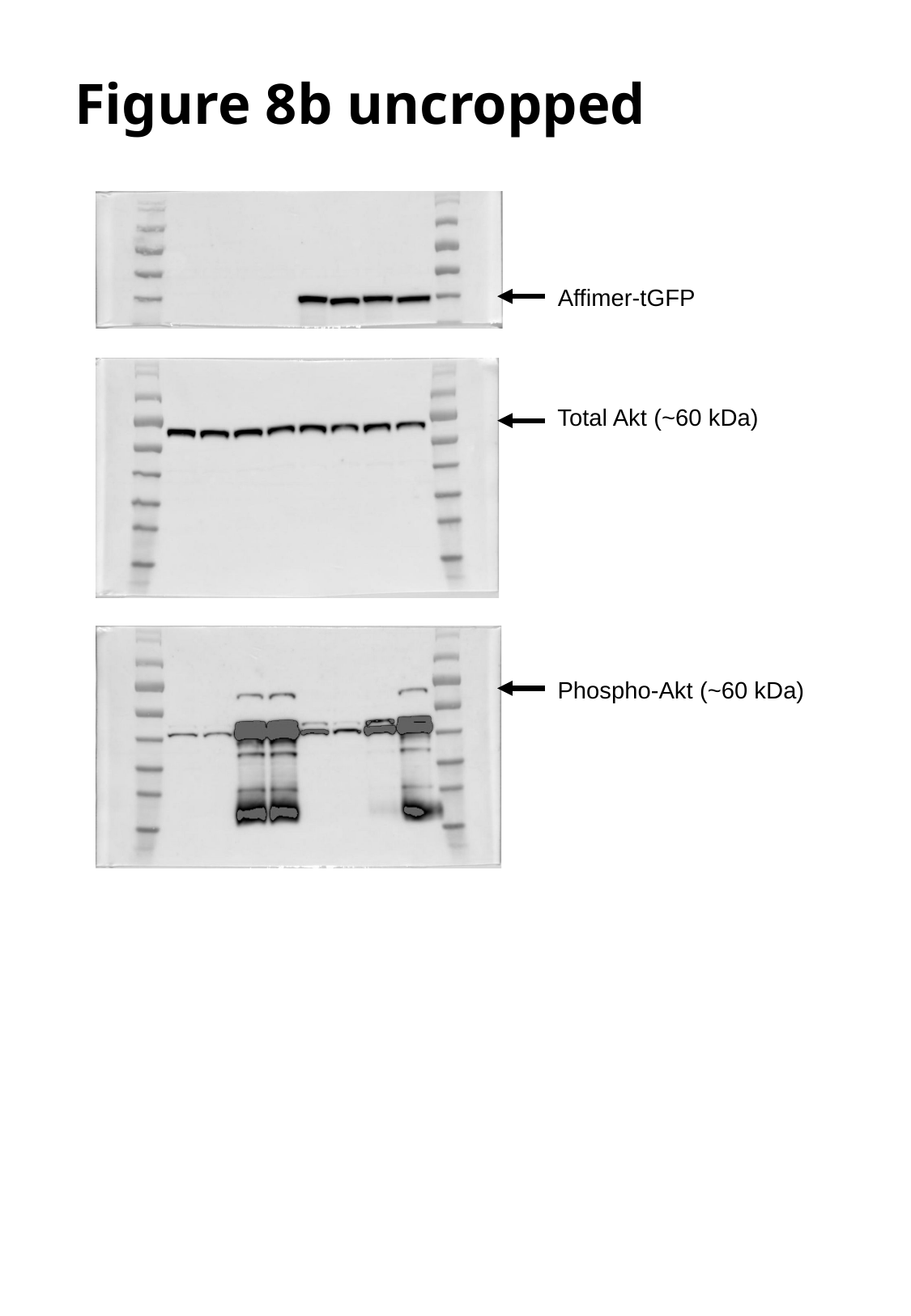

Figure 8b uncropped
Affimer-tGFP
Total Akt (~60 kDa)
Phospho-Akt (~60 kDa)
