# Supplementary material for: Targeting Grb2 SH3 Domains with Affimer Proteins Provides Novel Insights into Ras Signalling Modulation
Source: Biomolecules. 2024 Aug 22;14(8):1040. doi: 10.3390/biom14081040 (PMC11352564; doi:10.3390/biom14081040)
Supplement: Supplementary file 1 [file biomolecules-14-01040-s001.zip › Tang et al. Supplementary Materials.docx]

(a)


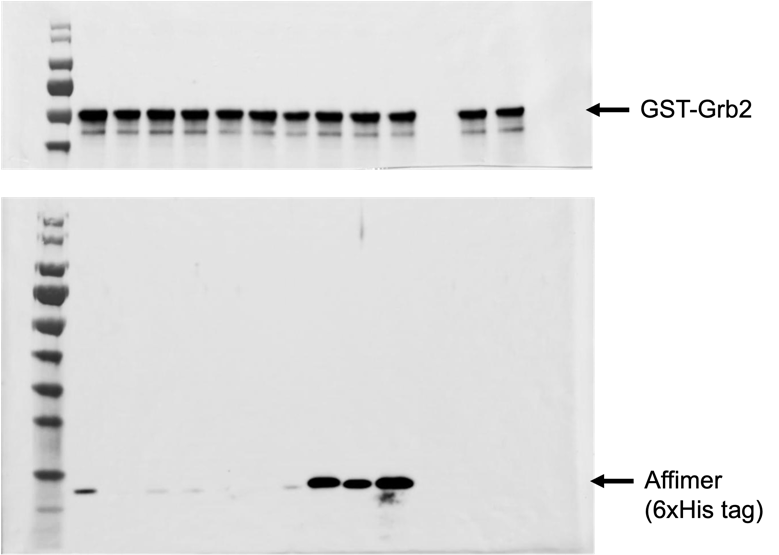


(b)


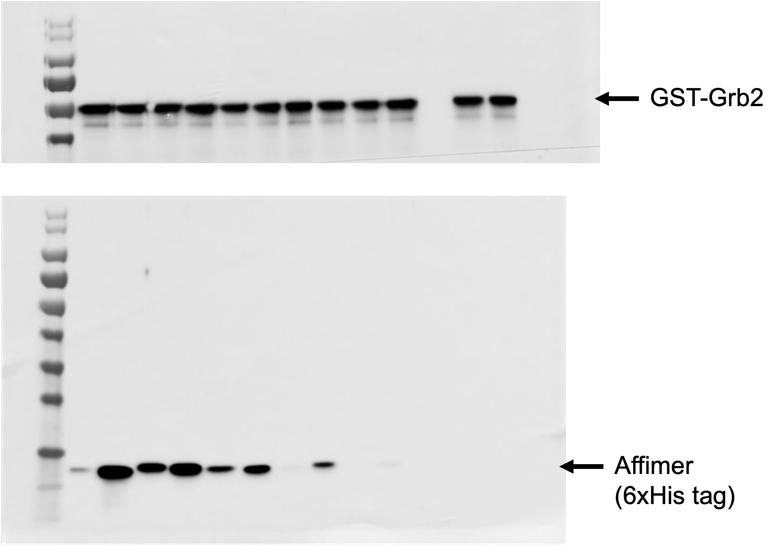


(c)


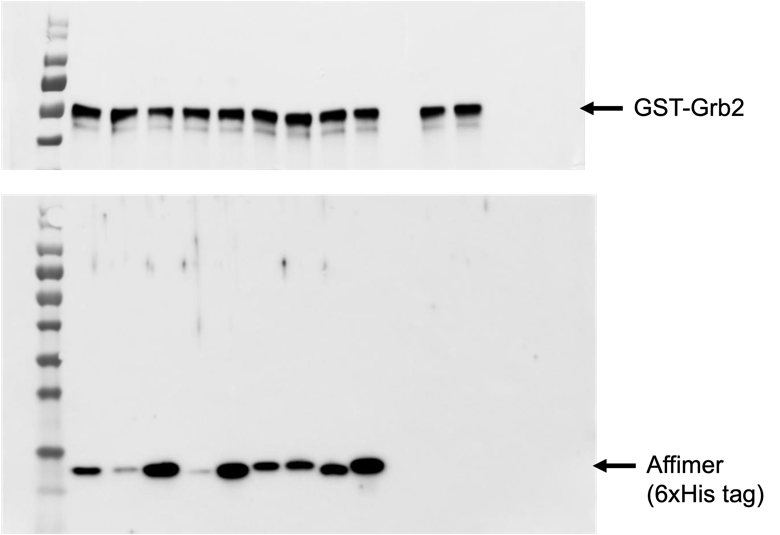


**Figure S1.** Uncropped Western blots for representative Figures 3a–c. Arrows denote regions of interest, as shown in the representative figures.

(a)


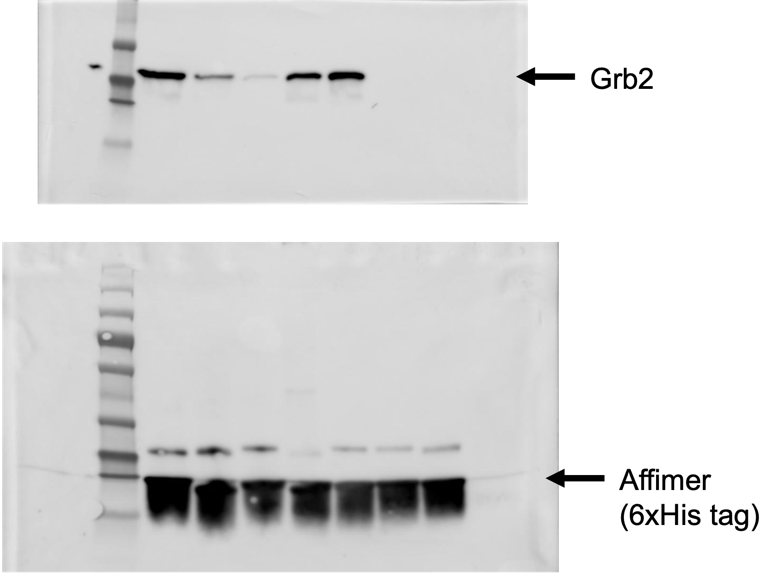


(b)


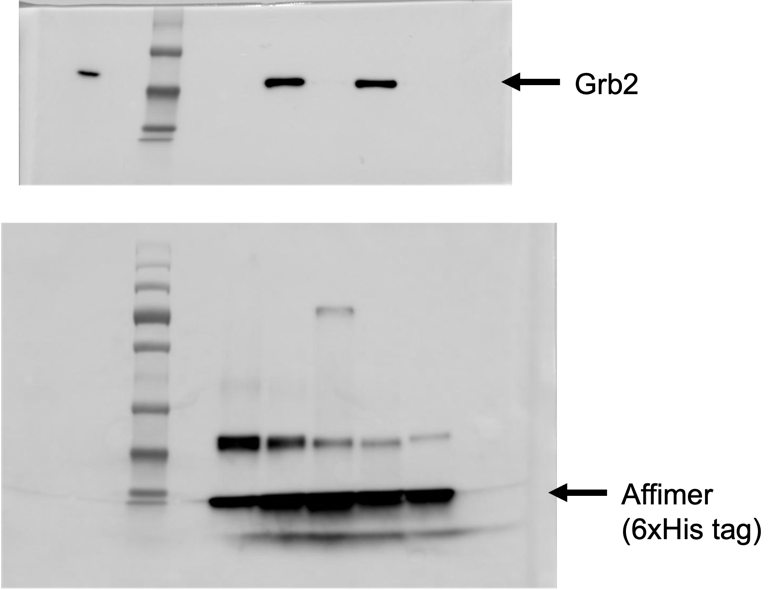


(c)


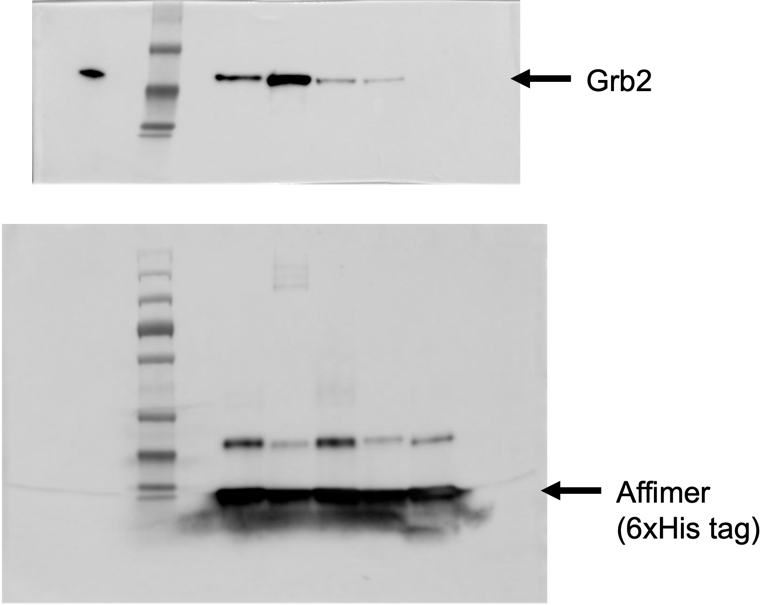


**Figure S2.** Uncropped Western blots for representative Figures 4a–c. Arrows denote regions of interest, as shown in the representative figures.

(a)


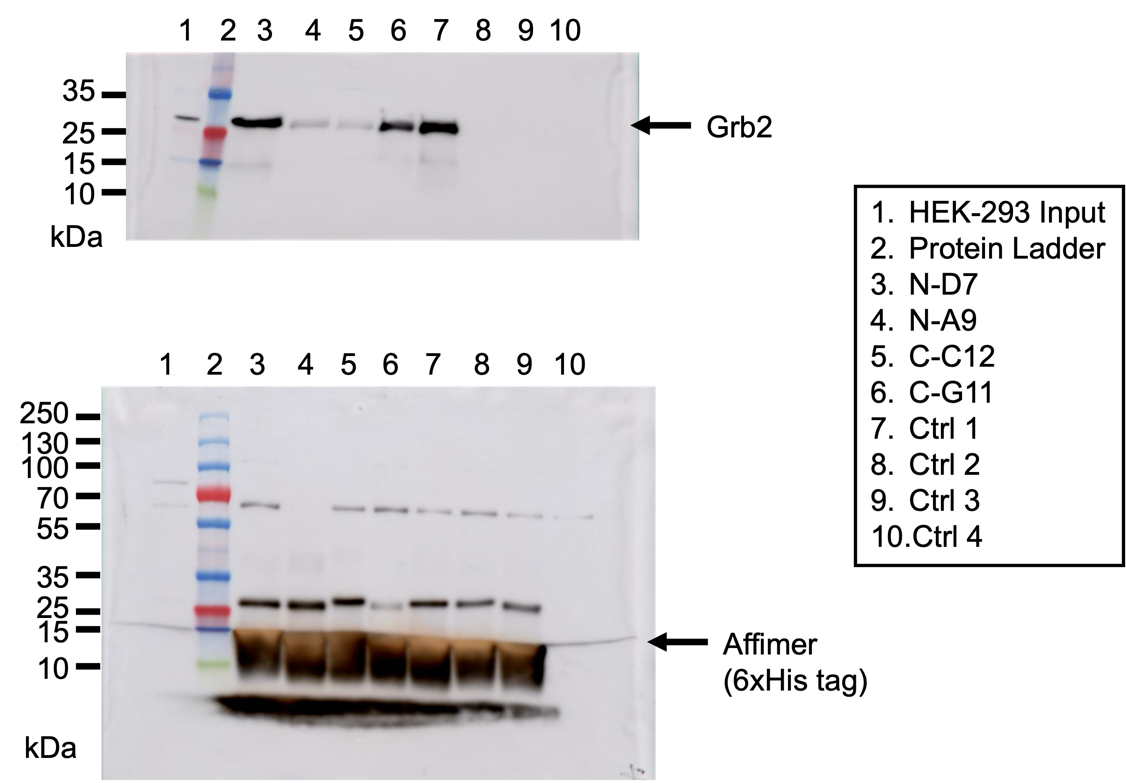


(b)


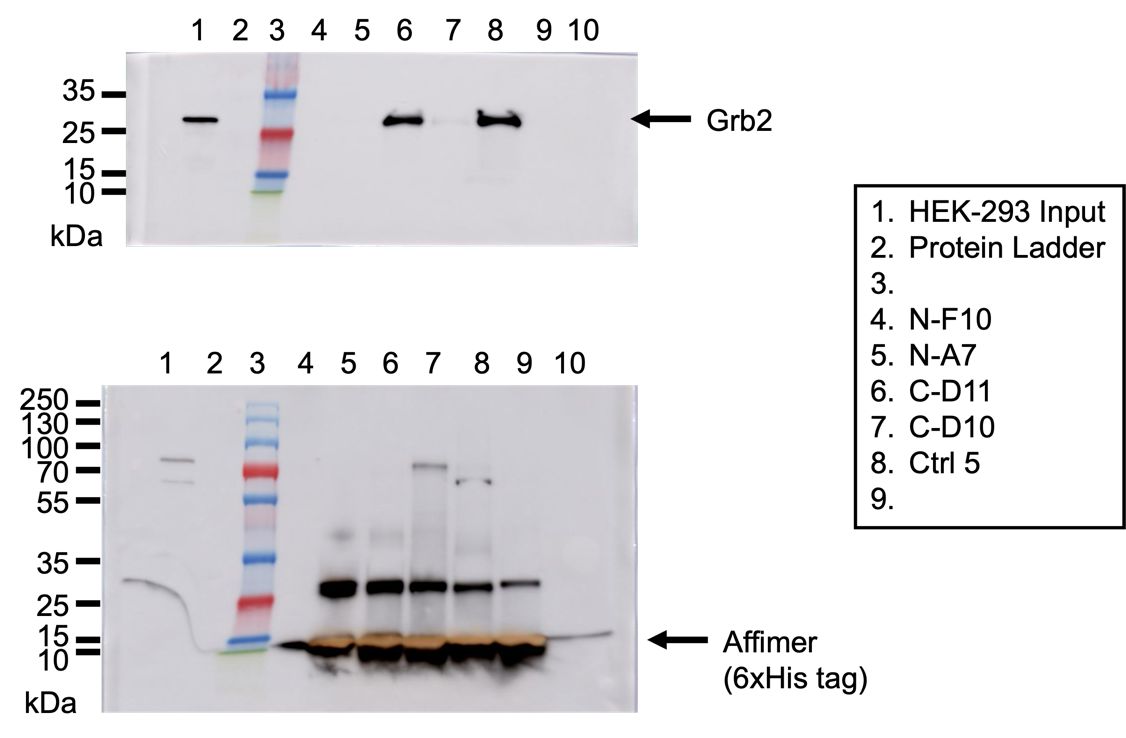


(c)


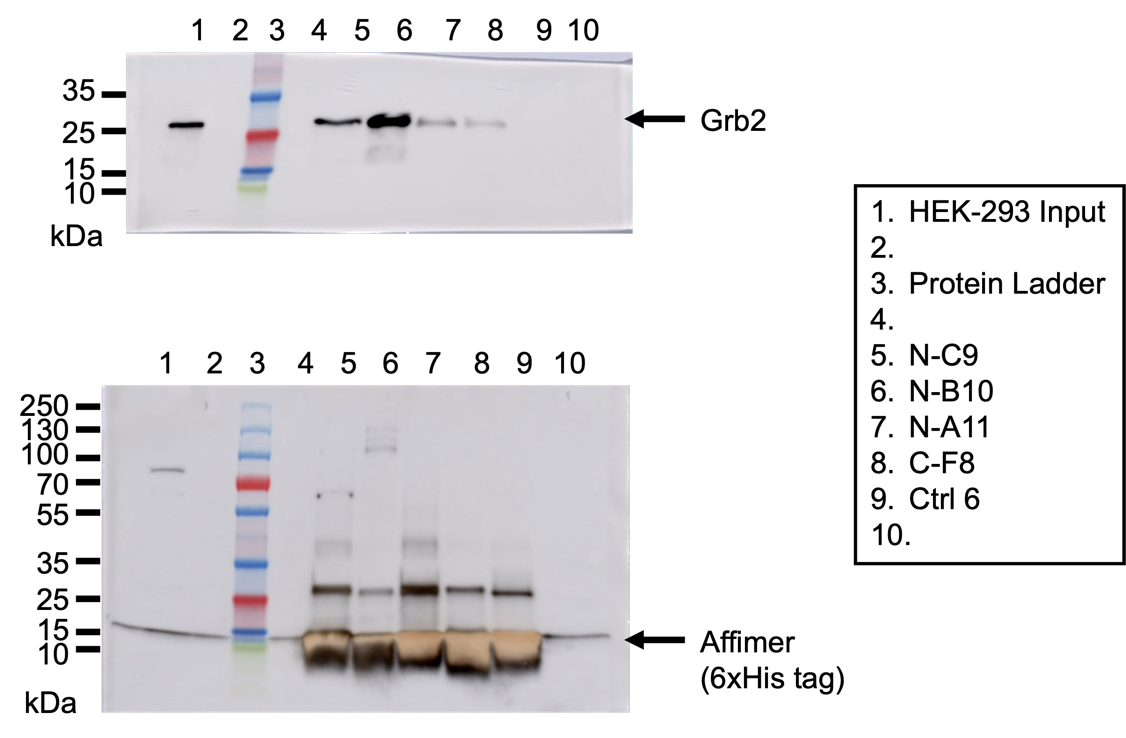


**Figure S3.** Representative anti-Grb2 and anti-6xHis tag uncropped Western blots from three independent experiments (n=3) demonstrating pull-down of endogenous Grb2 from HEK-293 cell lysates with immobilized 6xHis-tagged Affimer proteins. Samples were divided over three SDS-PAGE gels (a–c) with control samples loaded on each — ctrl 1 was a binder of the Grb2 SH2 domain; ctrl 2 was a binder of the PLCG1 SH3 domain; con-trol 3 was a non-Grb2 binding Affimer; ctrl 4 was a “no Affimer” control; ctrl 5 was another non-Grb2 binding Affimer and ctrl 6 was a “Affimer only” (no cell lysate) control.

(a)


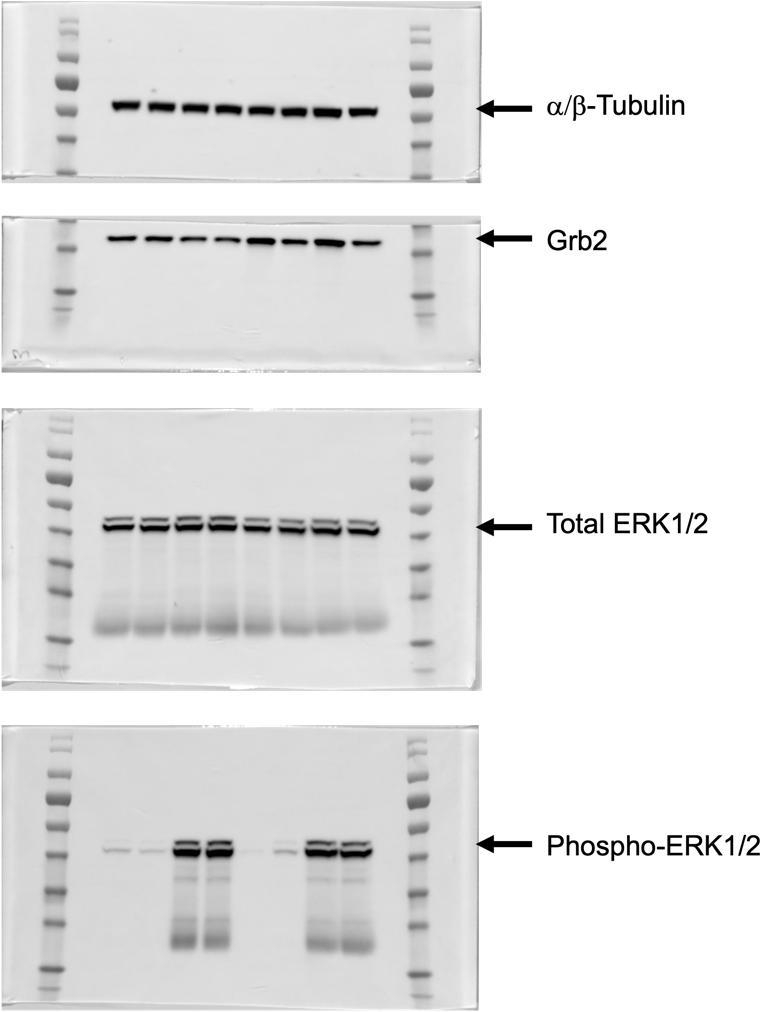


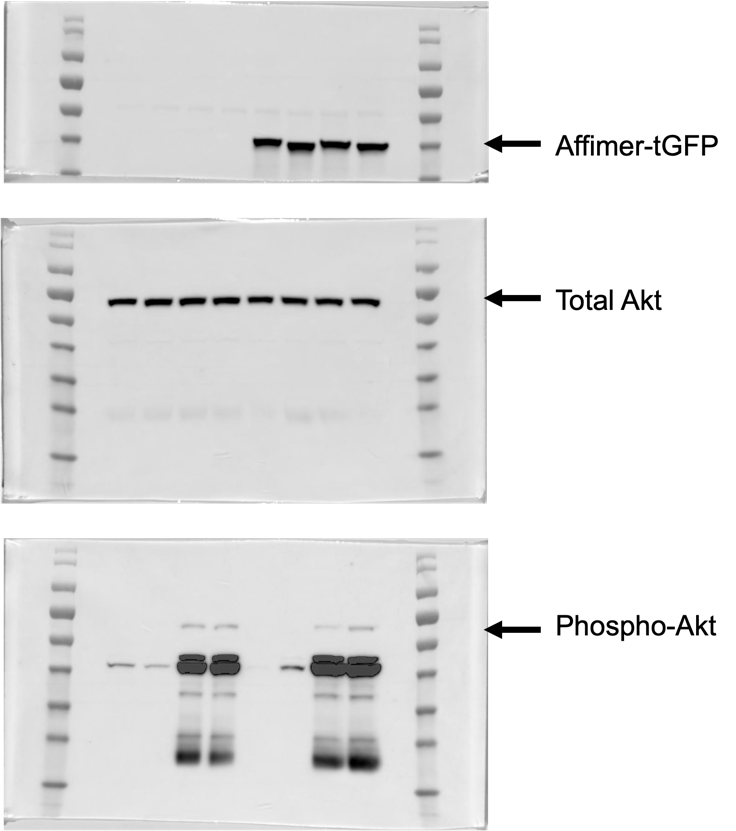


(b)


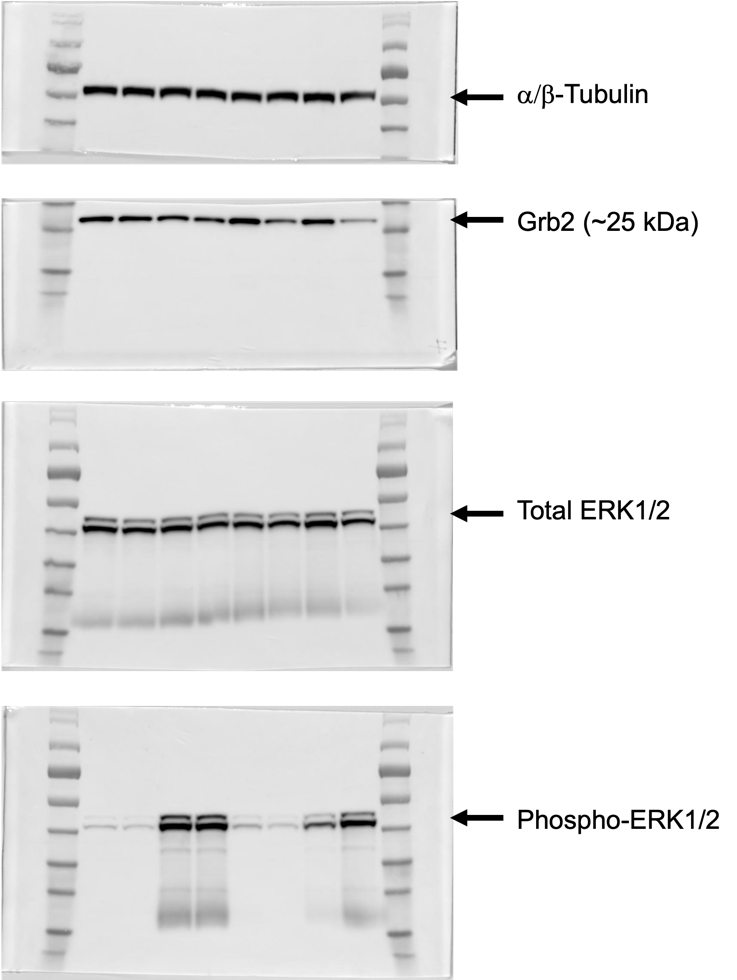


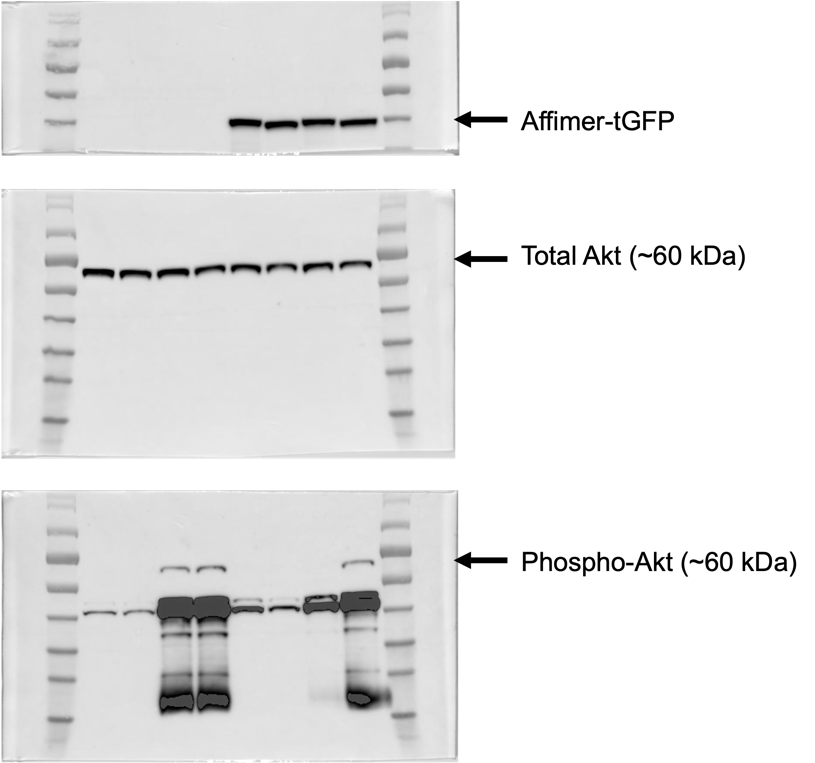


**Figure S4.** Uncropped Western blots for representative Figures 8a–b. Arrows denote regions of interest, as shown in the representative figures.
