# Supplementary figures and images for: Targeting Grb2 SH3 Domains with Affimer Proteins Provides Novel Insights into Ras Signalling Modulation
Source: Biomolecules. 2024 Aug 22;14(8):1040. doi: 10.3390/biom14081040 (PMC11352564; doi:10.3390/biom14081040)

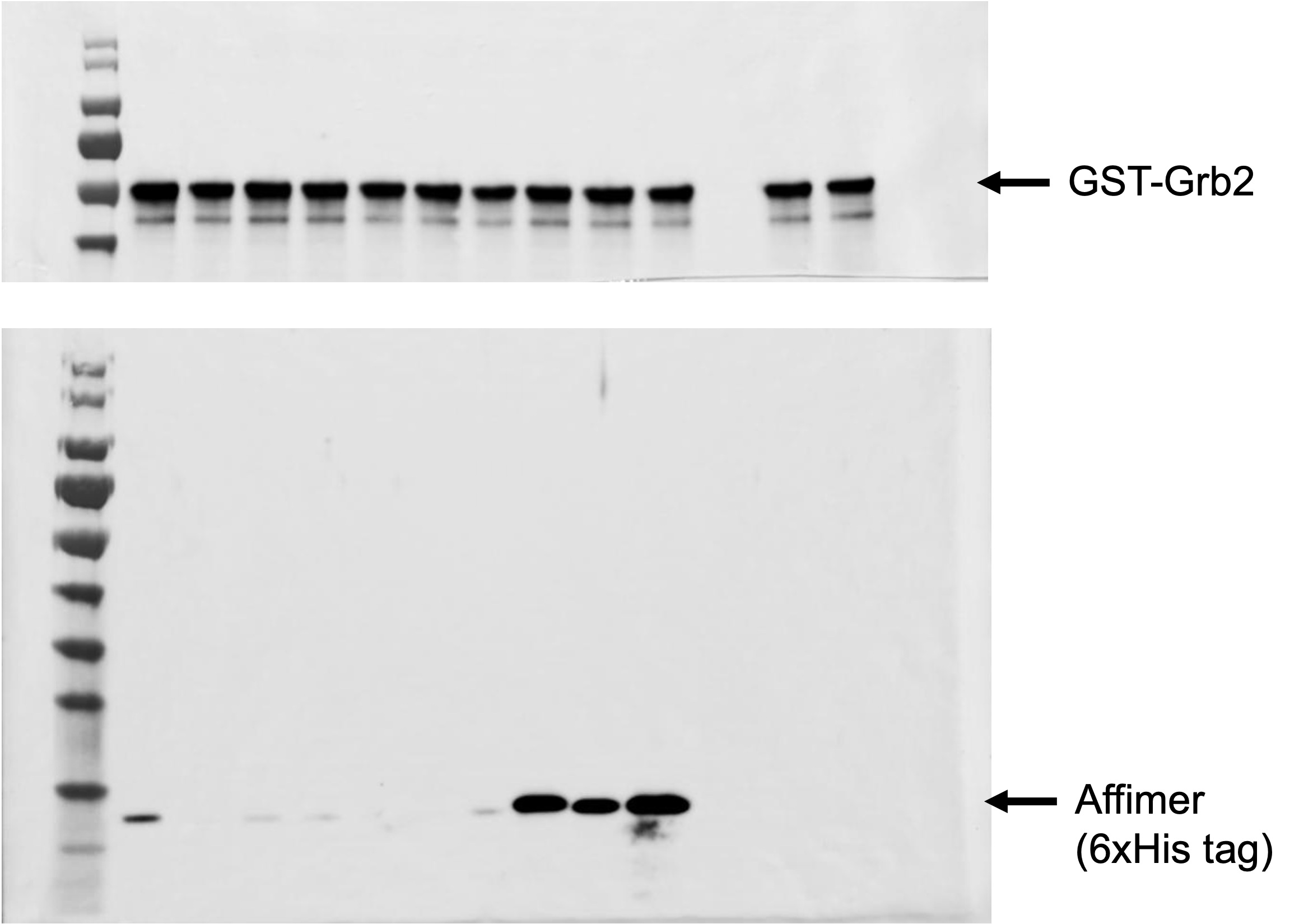

Supplement: Supplementary file 1 [file biomolecules-14-01040-s001.zip › Figure S1/Figure S1a.png]

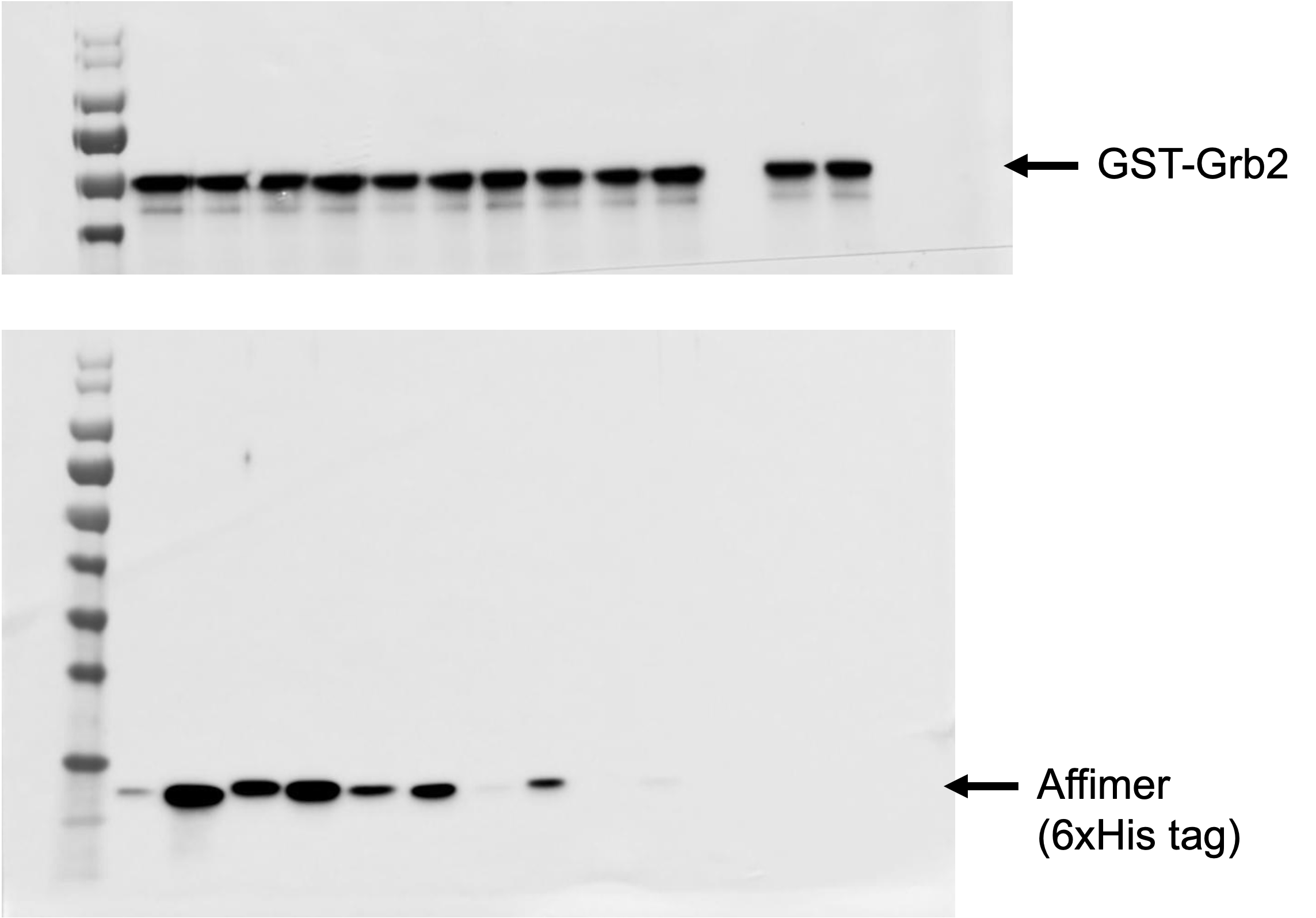

Supplement: Supplementary file 1 [file biomolecules-14-01040-s001.zip › Figure S1/Figure S1b.png]

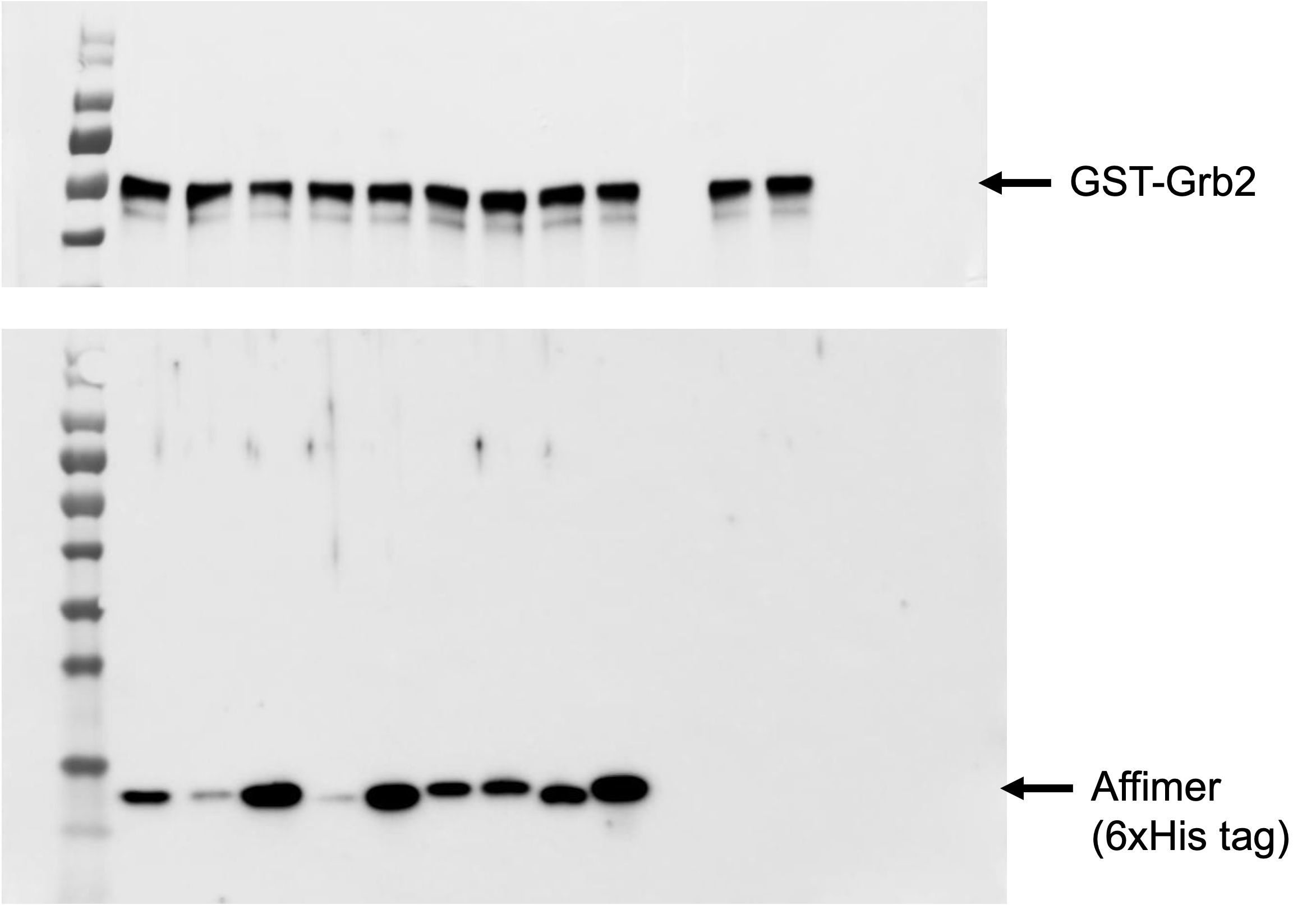

Supplement: Supplementary file 1 [file biomolecules-14-01040-s001.zip › Figure S1/Figure S1c.png]

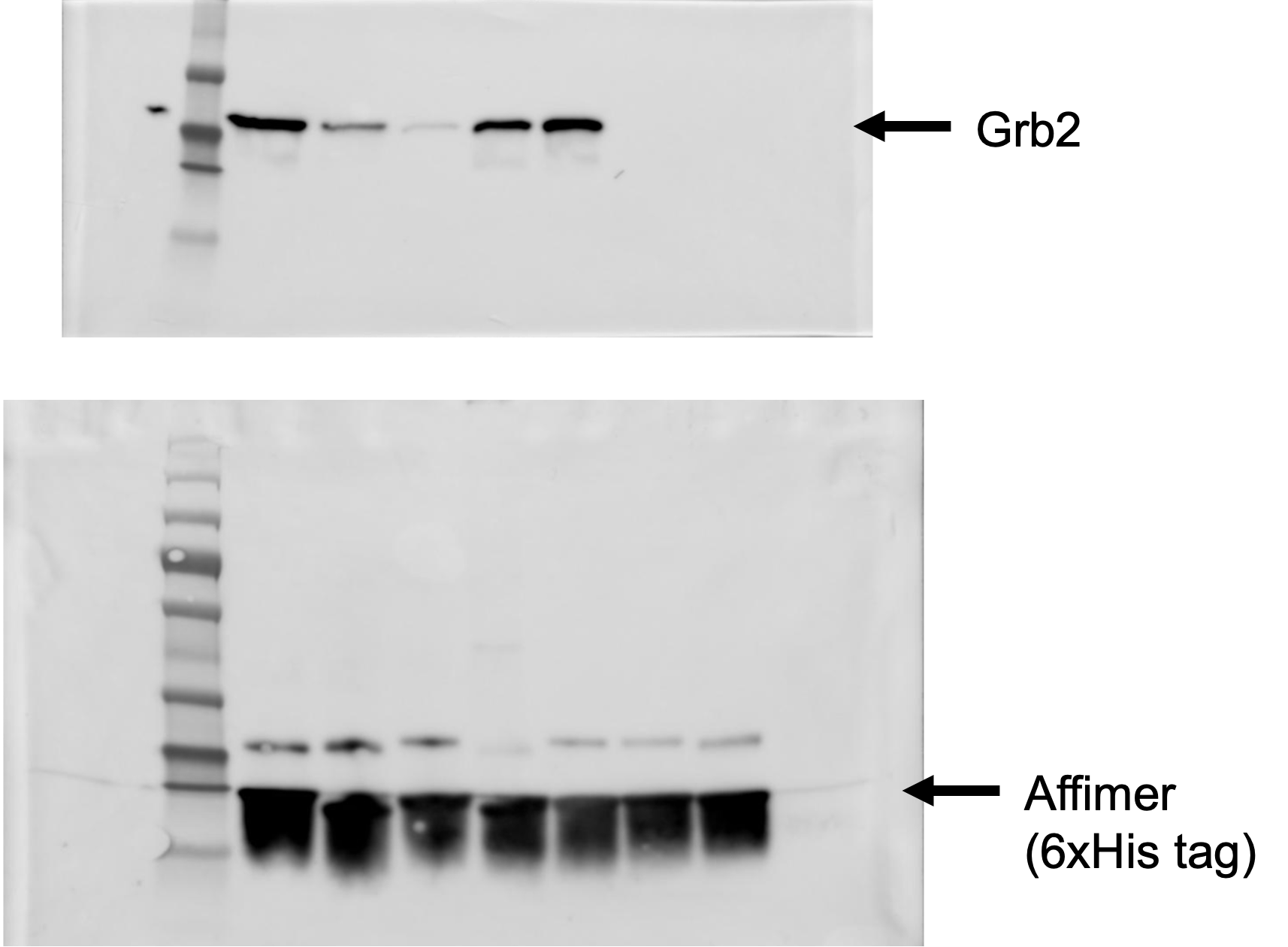

Supplement: Supplementary file 1 [file biomolecules-14-01040-s001.zip › Figure S2/Figure S2a.png]

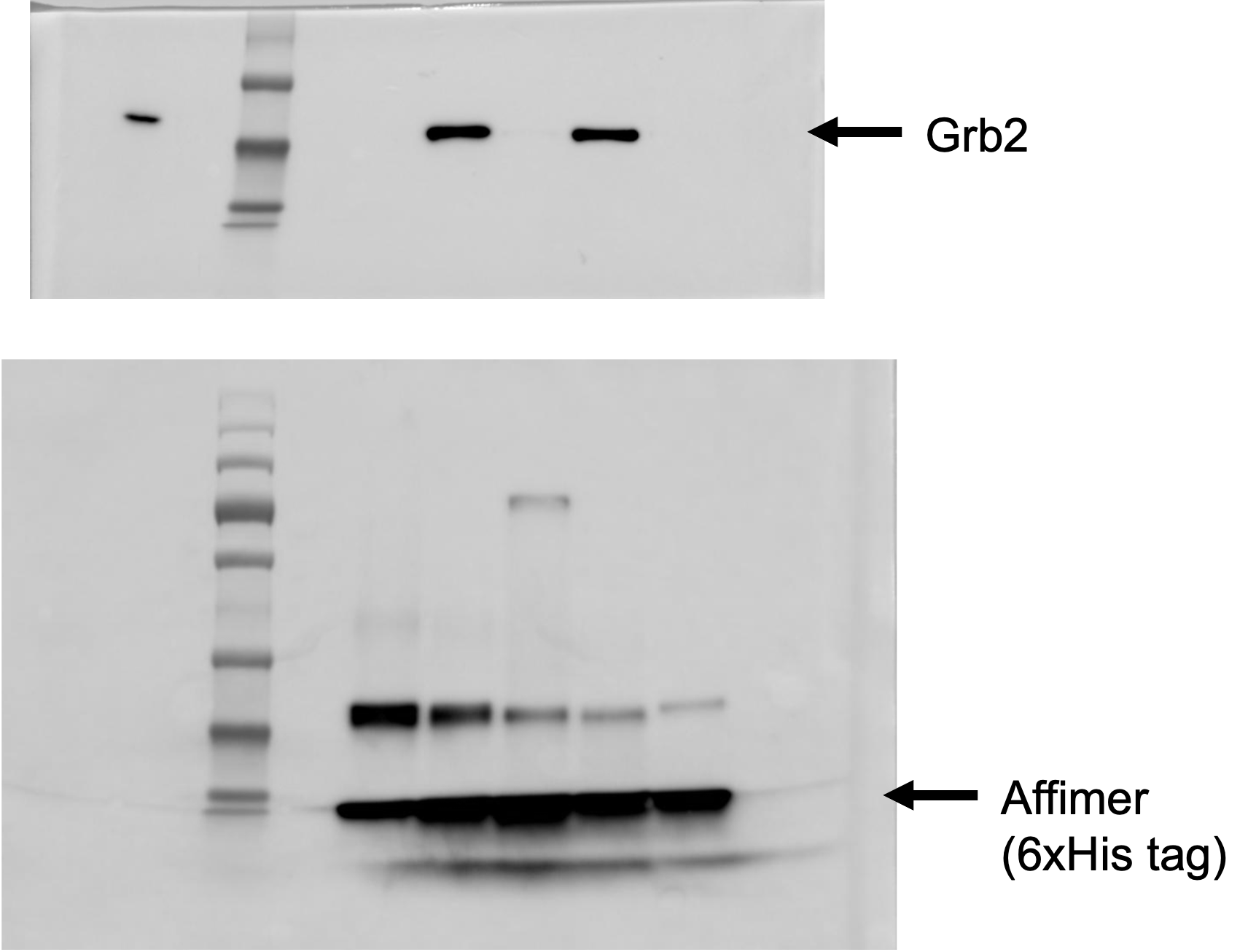

Supplement: Supplementary file 1 [file biomolecules-14-01040-s001.zip › Figure S2/Figure S2b.png]

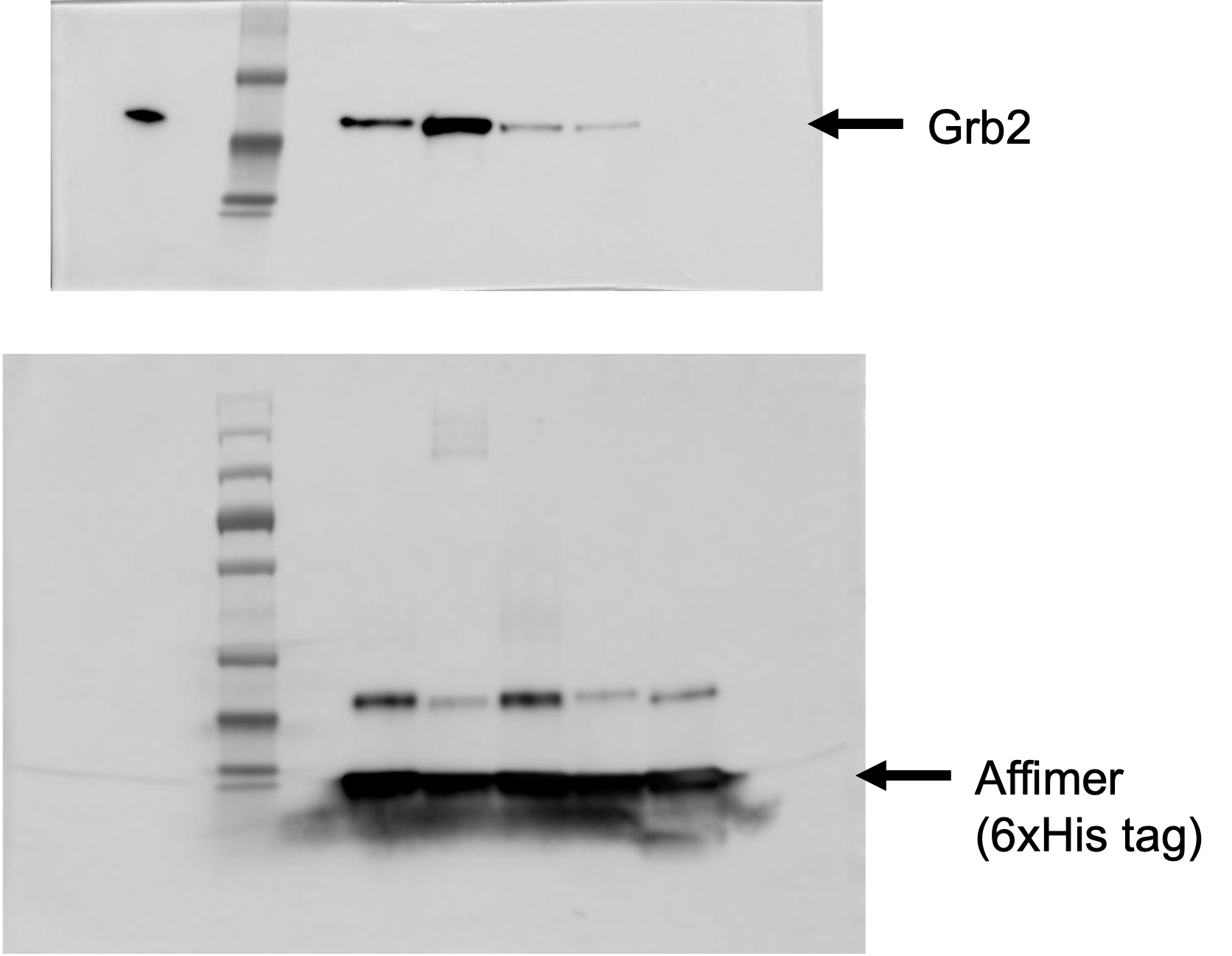

Supplement: Supplementary file 1 [file biomolecules-14-01040-s001.zip › Figure S2/Figure S2c.png]

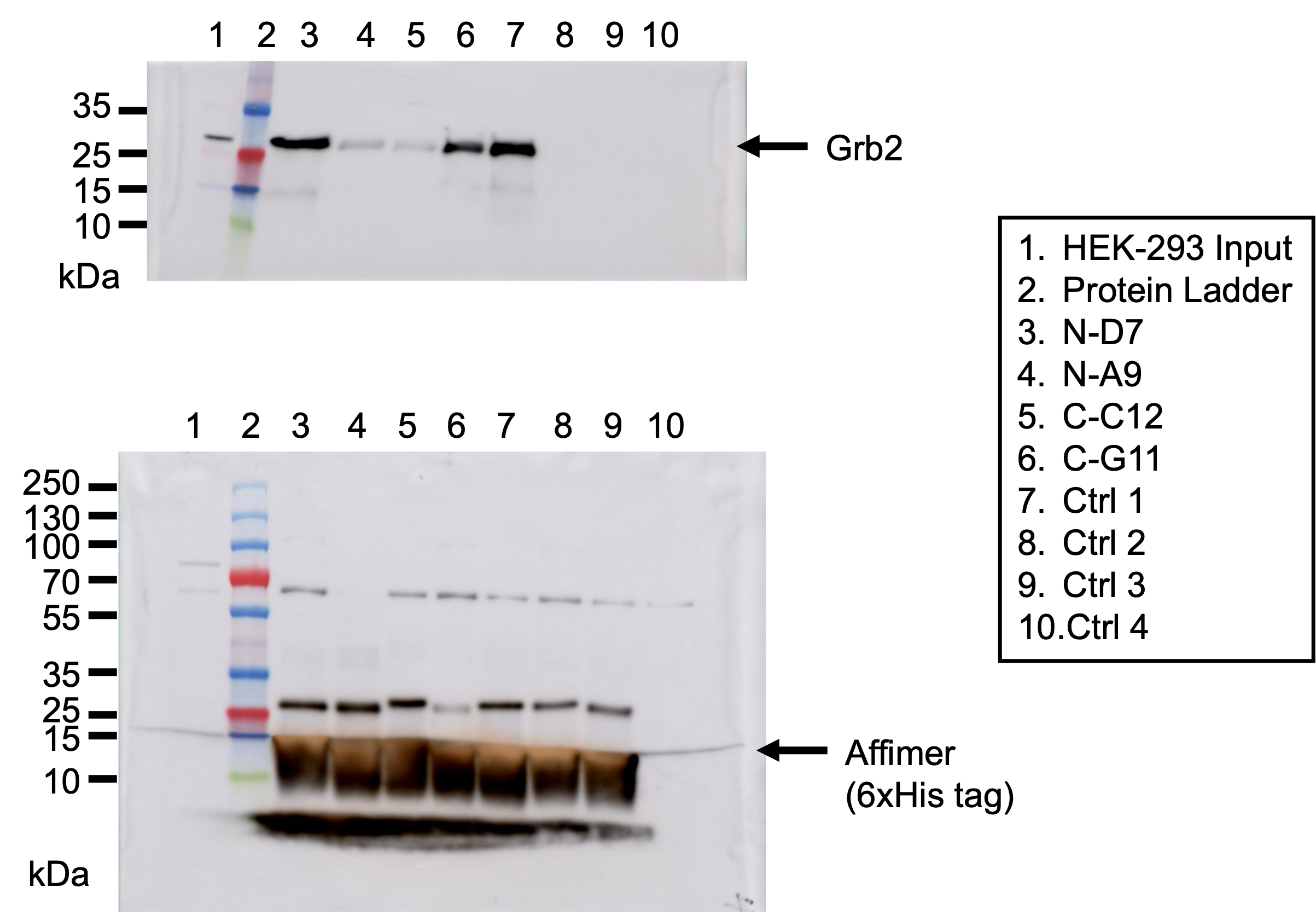

Supplement: Supplementary file 1 [file biomolecules-14-01040-s001.zip › Figure S3/Figure S3a.png]

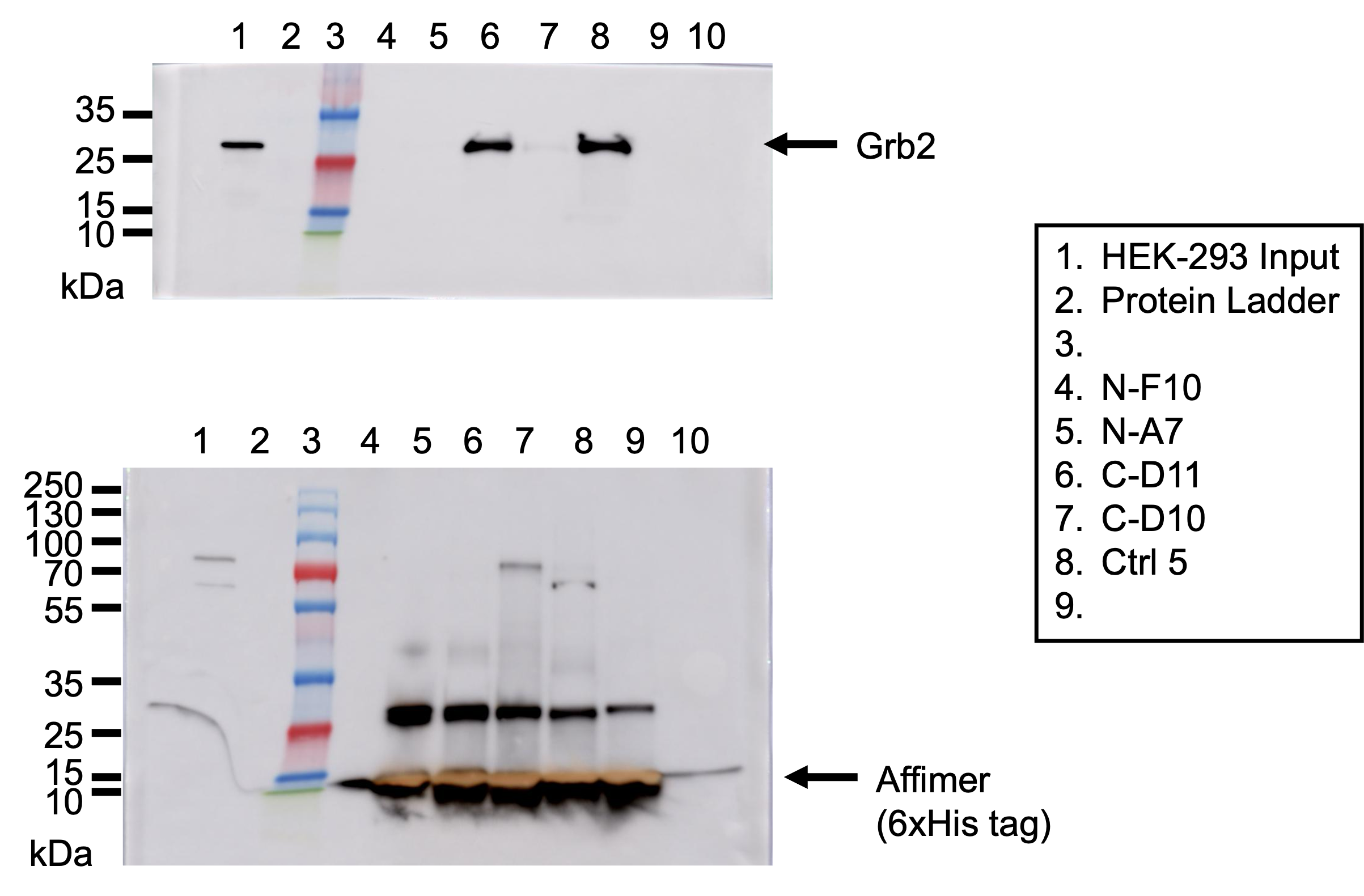

Supplement: Supplementary file 1 [file biomolecules-14-01040-s001.zip › Figure S3/Figure S3b.png]

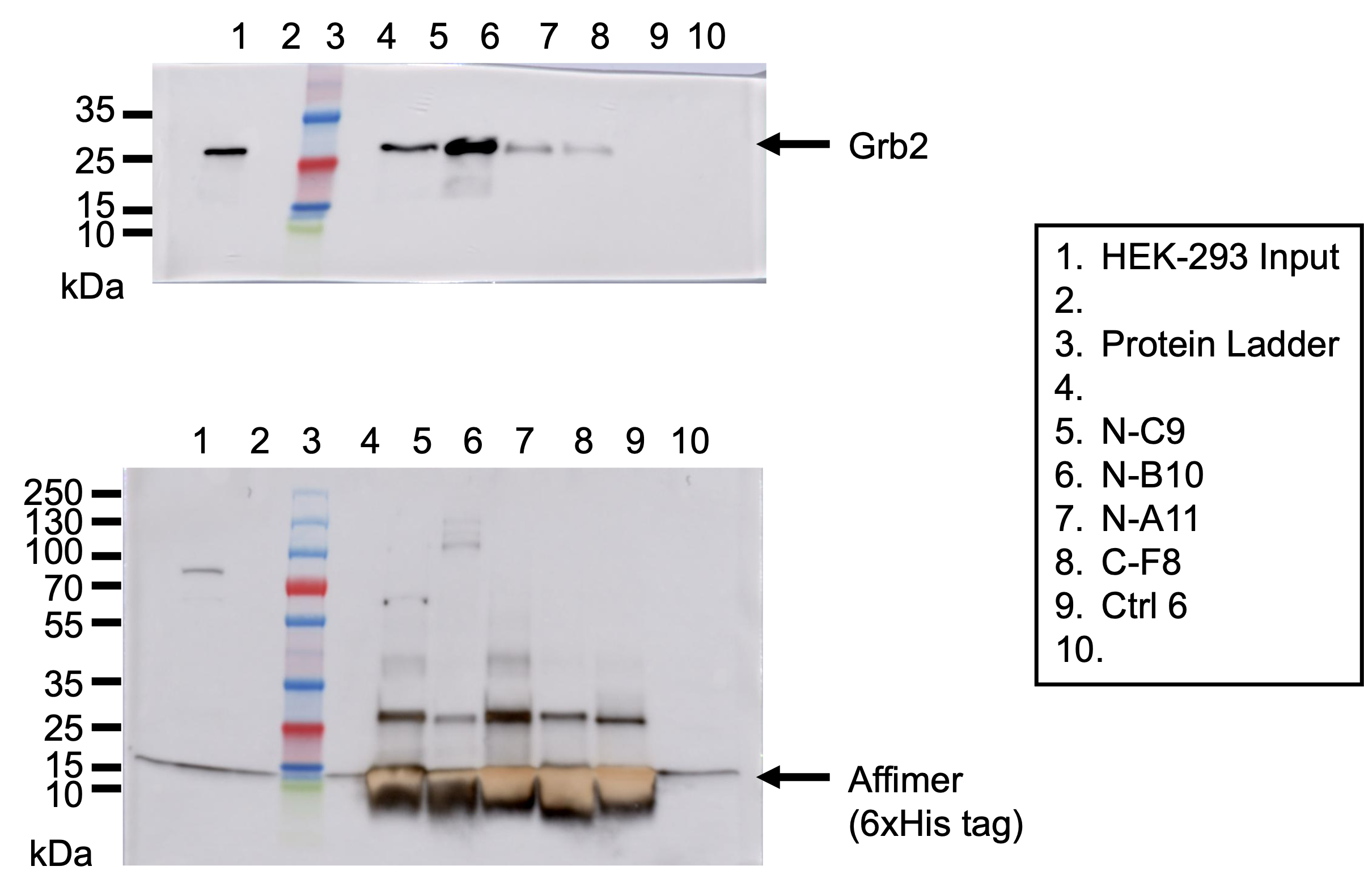

Supplement: Supplementary file 1 [file biomolecules-14-01040-s001.zip › Figure S3/Figure S3c.png]

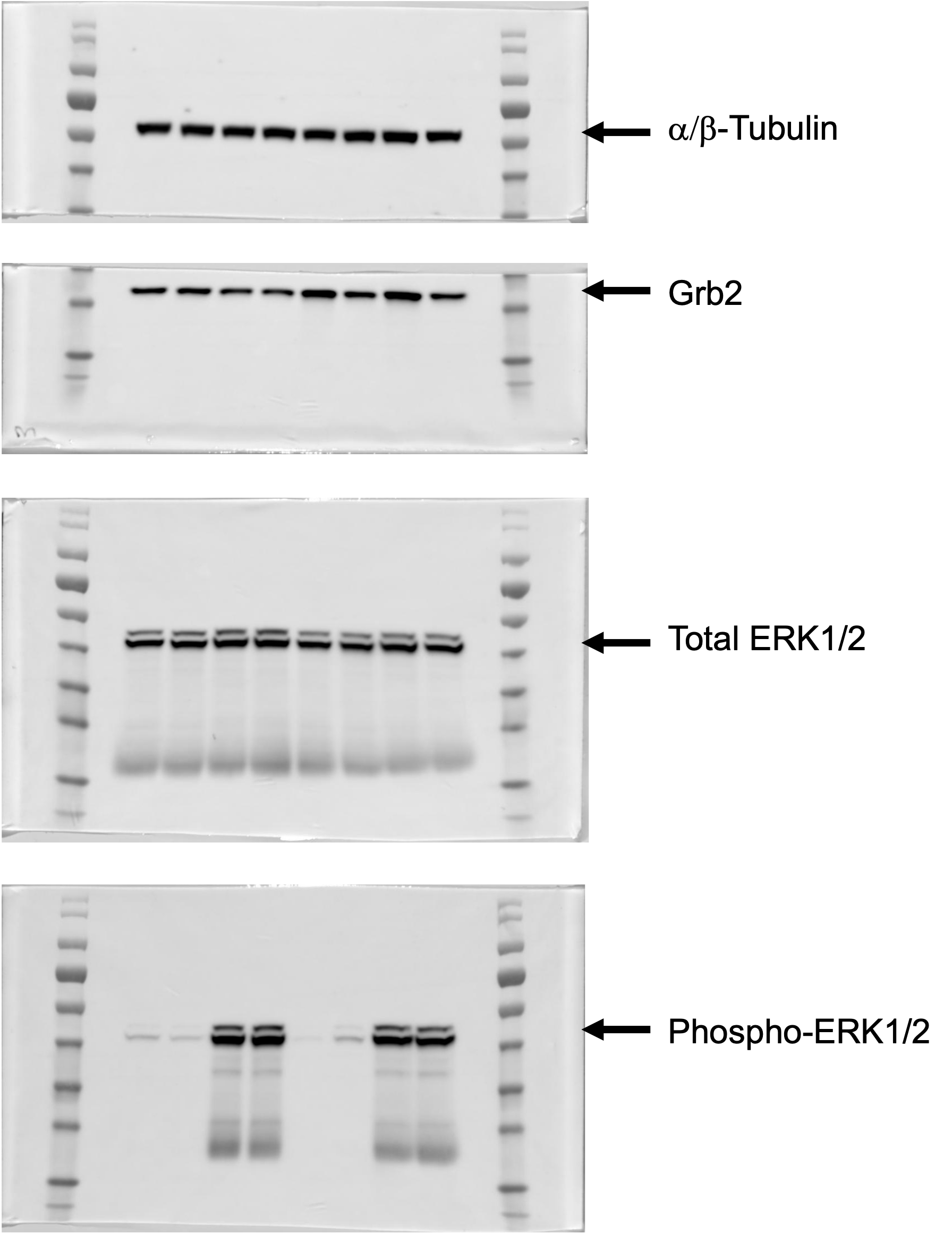

Supplement: Supplementary file 1 [file biomolecules-14-01040-s001.zip › Figure S4/Figure S4a (1).png]

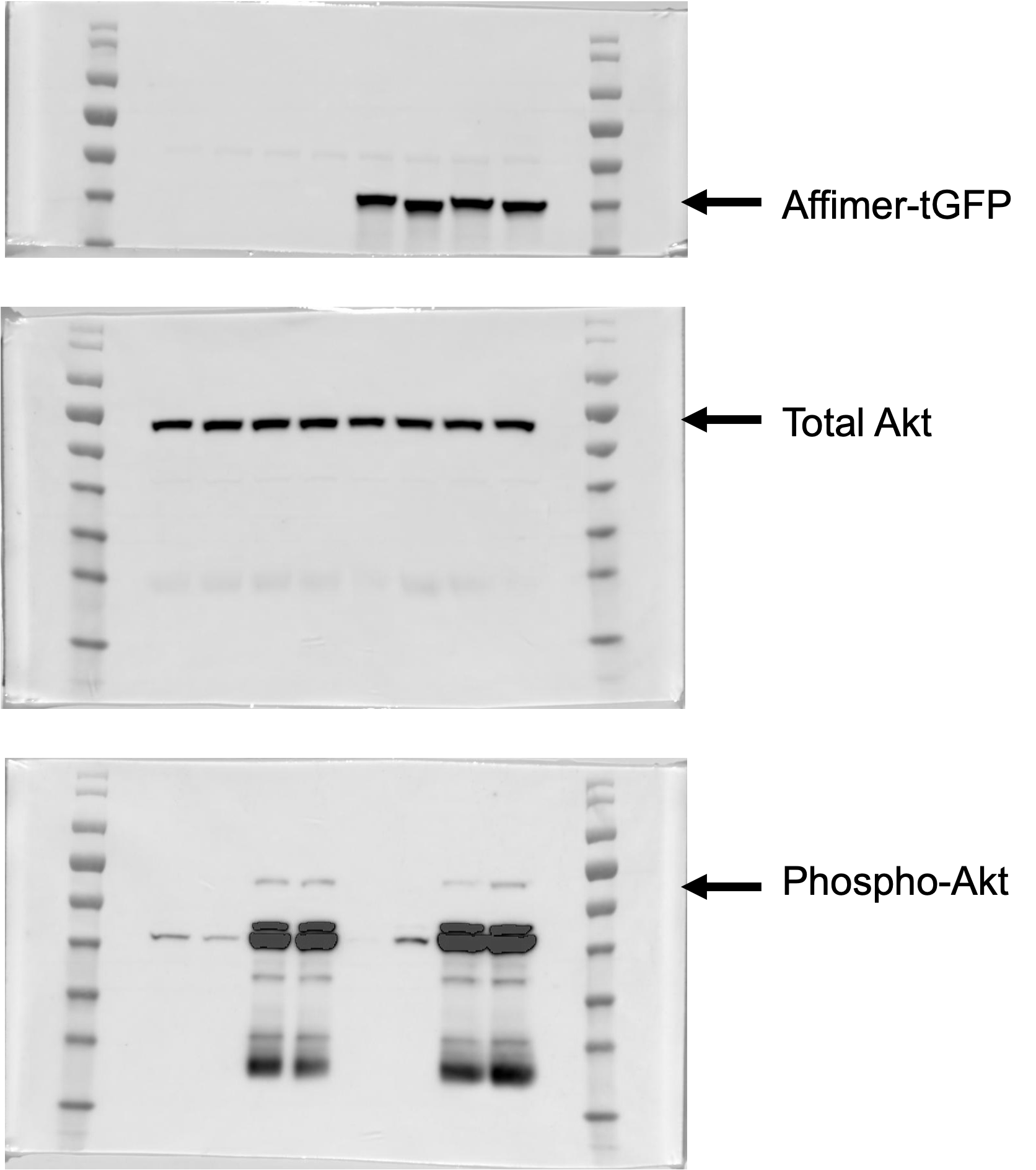

Supplement: Supplementary file 1 [file biomolecules-14-01040-s001.zip › Figure S4/Figure S4a (2).png]

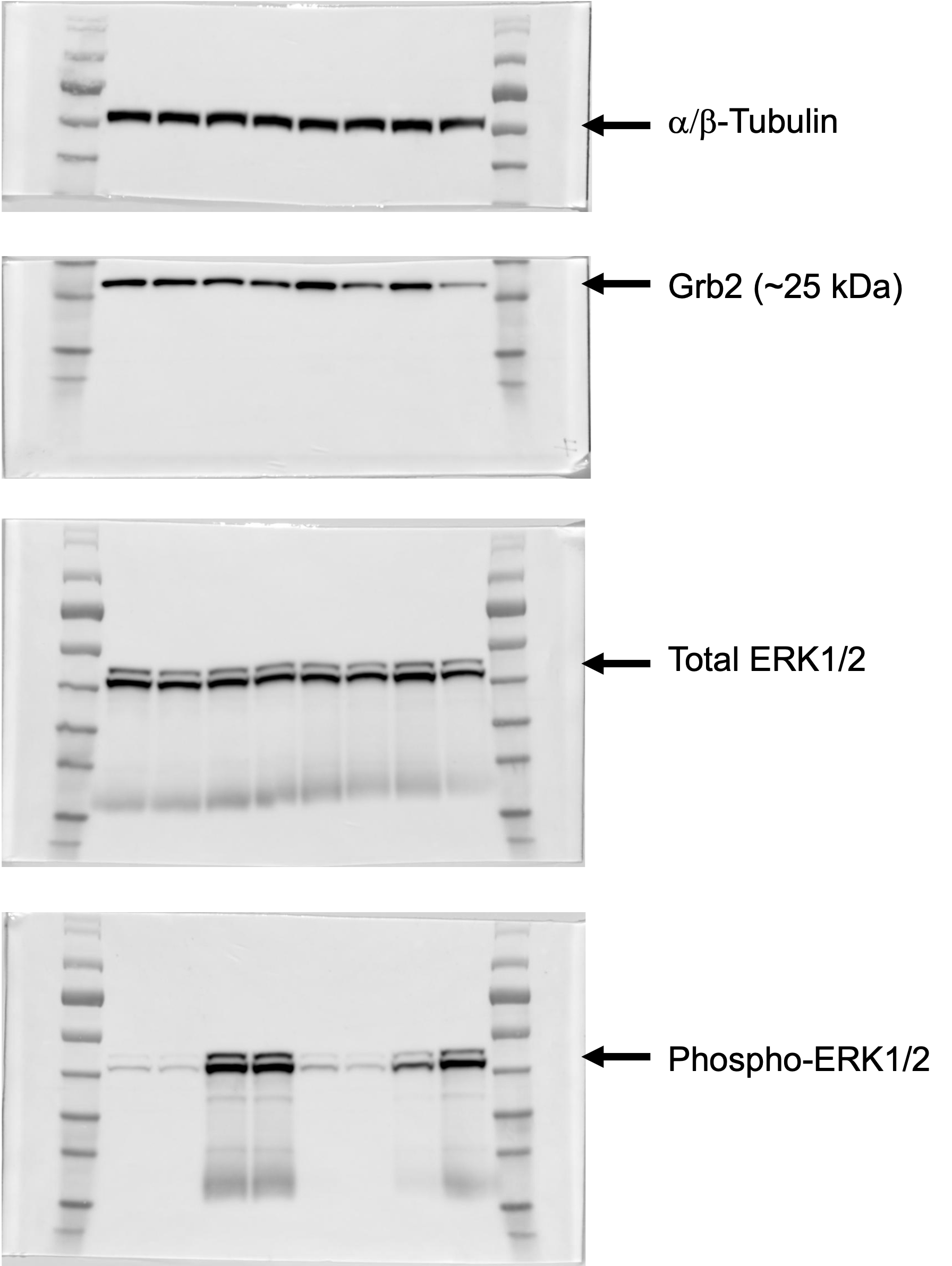

Supplement: Supplementary file 1 [file biomolecules-14-01040-s001.zip › Figure S4/Figure S4b (1).png]

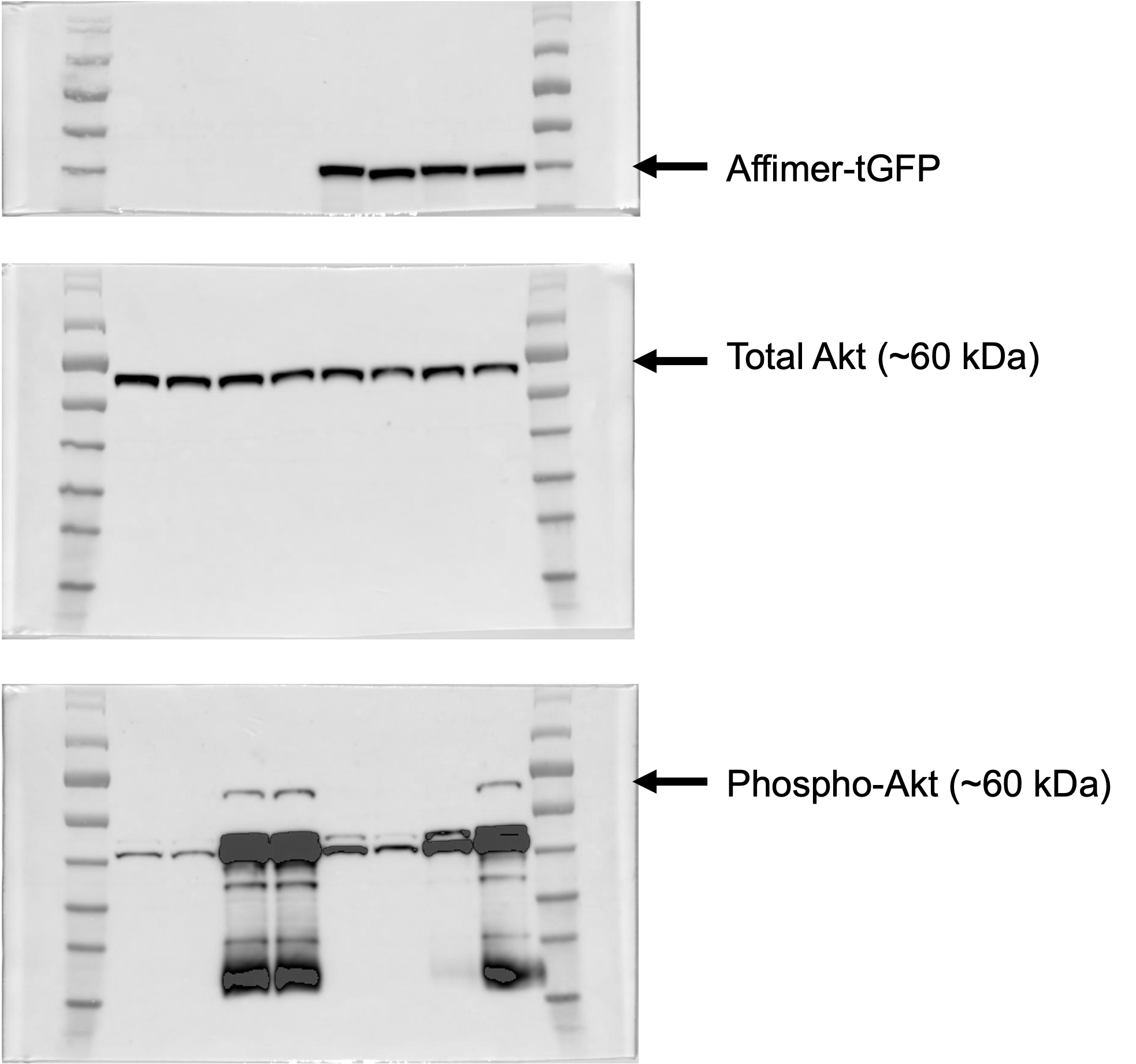

Supplement: Supplementary file 1 [file biomolecules-14-01040-s001.zip › Figure S4/Figure S4b (2).png]
